# Customizable tubular model for n-furcating blood vessels and its application to 3D reconstruction of the cerebrovascular system

**DOI:** 10.1007/s11517-022-02735-5

**Published:** 2023-01-26

**Authors:** Michal Chlebiej, Anna Zurada, Jerzy Gielecki, Mikolaj A. Pawlak, Maciej Szkulmowski

**Affiliations:** 1 Faculty of Mathematics and Computer Science, Nicolaus Copernicus University in Toruń, Chopina 12/18, 87-100 Torun, Poland; 2 Department of Radiology, Collegium Medicum, School of Medicine, University of Warmia and Mazury, Olsztyn, Poland; 3 Department of Anatomy, Collegium Medicum, University of Warmia and Mazury, Olsztyn, Poland; 4 Department of Neurology and Cerebrovascular Disorders, Poznan University of Medical Sciences, Fredry 10, 61-701, Poznan, Poland; 5 Department of Clinical Genetics, Erasmus MC, PO Box 2040, 3000 CA Rotterdam, The Netherlands; 6 Faculty of Physics, Astronomy and Informatics, Nicolaus Copernicus University in Toruń, Grudziadzka 5, 87-100 Torun, Poland

**Keywords:** Computer-aided detection and diagnosis, Magnetic resonance imaging (MRI), Cerebral vessels segmentation

## Abstract

Understanding the 3D cerebral vascular network is one of the pressing issues impacting the diagnostics of various systemic disorders and is helpful in clinical therapeutic strategies. Unfortunately, the existing software in the radiological workstation does not meet the expectations of radiologists who require a computerized system for detailed, quantitative analysis of the human cerebrovascular system in 3D and a standardized geometric description of its components. In this study, we show a method that uses 3D image data from magnetic resonance imaging with contrast to create a geometrical reconstruction of the vessels and a parametric description of the reconstructed segments of the vessels. First, the method isolates the vascular system using controlled morphological growing and performs skeleton extraction and optimization. Then, around the optimized skeleton branches, it creates tubular objects optimized for quality and accuracy of matching with the originally isolated vascular data. Finally, it optimizes the joints on n-furcating vessel segments. As a result, the algorithm gives a complete description of shape, position in space, position relative to other segments, and other anatomical structures of each cerebrovascular system segment. Our method is highly customizable and in principle allows reconstructing vascular structures from any 2D or 3D data. The algorithm solves shortcomings of currently available methods including failures to reconstruct the vessel mesh in the proximity of junctions and is free of mesh collisions in high curvature vessels. It also introduces a number of optimizations in the vessel skeletonization leading to a more smooth and more accurate model of the vessel network. We have tested the method on 20 datasets from the public magnetic resonance angiography image database and show that the method allows for repeatable and robust segmentation of the vessel network and allows to compute vascular lateralization indices.

**Graphical abstract:**

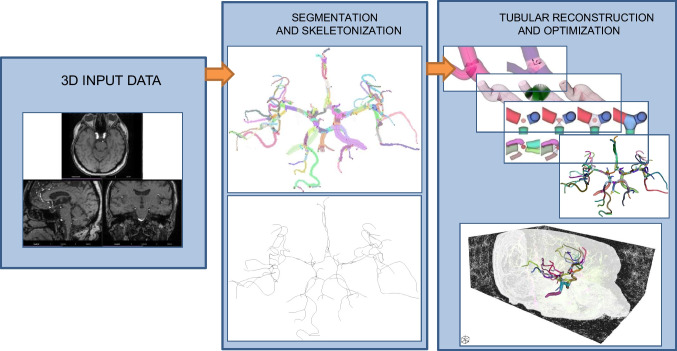

## Introduction

Identification of vascular structures is one of the most critical clinical targets in brain imaging. In clinical practice, the role of vascular imaging has been growing exponentially over the last 3 decades. The reason for this is the epidemic of brain diseases that are either directly or indirectly influencing the cerebral vessels. Ischemic stroke directly affects the cerebral vessels by mechanically occluding the vessels in the brain and impairs the flow, resulting in tissue death as well as clinical symptoms associated with the affected region of the brain. In 2016, it contributed to 5.5 million deaths and 116.4 million disability-adjusted life years (DALY) worldwide [22]. Stroke is often associated with an indirect vascular injury caused by diabetes in which a continued high concentration of blood glucose impairs the function of the small vessels, leading to an increase in the mortality and morbidity of all patients affected by this common condition. The steady growth of cerebrovascular risk factors and the number of patients suffering from chronic diseases injuring cerebral vessels require the development of reliable and available methods for quantitative assessment of the vascular structures to be ideally performed in a fully automatic manner. The rise of mechanical thrombectomy shows how the stroke outcome caused by rapid identification of large cerebral vessel occlusion (LVO) can improve by using novel therapeutic approaches that depend on precise image-based diagnosis [6]. LVO is identified in small hospitals that often do not have the expertise and experience to treat such patients. Remote and computer-assisted diagnosis systems enable physicians to quickly and safely diagnose based on imaging features derived from computed tomography angiography (CTA) and magnetic resonance angiography (MRA). Both methods enable visualization of the cerebral vessels. However, the basic form does not provide quantitative measures of the cerebral vascular network. We aim at providing a tool that enables automatic vessel segmentation, detects vessel divisions, and includes lumen quantification of the cerebral vascular system.

Vascular segmentation and reconstruction methods fall into two categories—voxel-based methods and machine-learning methods. A comprehensive review of such techniques has been performed [24]. Voxel-based methods are the most common. Antiga et al*.* generated patient-specific vessel meshes for computational fluid dynamics analysis by using a level set [1, 33]. Wang et al*.* proposed the integrated level set method for boundary detection using iteratively refining centerlines [43]. A different approach was presented by Shahzad et al*.* who segmented the lumen by combining graph cuts and kernel regression to detect stenosis accurately [37]. Reference [31] presented a method for the reconstruction of human cerebrovasculature based on 3D MR data. The technique used modeling of the vasculature based on tubular reconstruction using centerline detection, radius estimation, and bifurcation joints reconstruction. Several open-source libraries such as VMTK [33] and TubeTK [4] have been developed, and they provide API functions for implementing some vessel segmentations.

In VMTK, centerlines are determined as the paths defined on Voronoi diagram sheets. The goal is to minimize the integral of the radius of maximal inscribed spheres along the path. Such an approach leads to finding the shortest paths in the radius metric. Detailed problem-dependent customization vascular tree, i.e., detection/removal of short leaves and inner segments and or segments defined by custom geometrical properties. VMTK operates in two modes—global reconstructing complete vascular tree using gradient-based 3D level set segmentation or interactive mode searching for the optimal path between two points (single vascular segment). TubeTK library enables tube segmentation based on centerline extraction with emphasis on image filtering and edge enhancement utilizing features of the ITK library [27].

The above voxel-based methods are capable of handling bifurcation geometry and constructing seamless modes. None of the above-mentioned libraries give a straightforward possibility for customization of skeleton optimization/simplification, definition, and parameterization of joint segments.

The machine learning approach for the segmentation methods can be computationally more efficient and learn from manually labeled ground truth models. Reference [25] used the probabilistic boosting tree (PBT) to detect vessel lumen boundary by training the PBT classifier from manually labeled ground truths presented in [3]. As stated by [46], the mesh should be watertight, of high quality, and should have no mesh intersection. Fulfilling these criteria in machine learning approaches is still a challenging task. Zhou et al*.* presented a solution for coronary artery segmentation [46]. The methods for automatic segmentation and labeling are mainly used for specific vascular segments and are limited to a set of a few simple bifurcations. Authors [8] generate tubular trees based on region-growing segmentation and thinning for skeletonization. The main goal of this work is the automation of the labeling process. In [35], the authors segment an image using the cascade of filters in a multiresolution manner, calculating probabilities of voxel belonging to the center of a vessel of a given radius. The method operates globally calculating probabilities in different image resolutions. A designed classifier based on all multiscale decisions generates final classification if source voxel belongs to a vessel. The last part searches for probability maximum values and forms the directed graph representing vascular tree defined by skeletal segments and radius values. This method, like many others, does not treat separately volumetric joints of connecting tubes—they may overlap. The authors only mention the problem. The main focus in this work is on automatic labeling of reconstructed objects. In [39], the authors define prior shapes base model and neural networks for automatic generation of vascular reconstruction. Despite the advantage of automation, the method strongly relies on the initial shape database and cannot be interactively customized. Such an approach can be crucial for pathology cases.

In this study, we focus on solving the problems that we encountered in our studies when using the abovementioned approaches. In particular, for vessel segmentation immune to local intensity fluctuations, we use a voxel-based approach based on the assumptions made by Nowinski et al. [31] that is combined with a number of novel solutions for (1) customized skeleton tracing and optimization, (2) optimized generation of tubular segments of vessels, (3) removal of collisions and self-collisions in the mesh of generated vessels, and (4) reconstruction of vessel mesh in the vessel joints, where the vessel divides into n smaller vessels (n-furcations). An additional benefit of the method is the full control of the process of vessel network growth with a parametric selection of vessel properties entering the growth model and, as a consequence, access to predecessors and successors of each vessel segment allowing the quantitative analysis of the vessel network as a graph with or without loops. This allows us to extract a number of simple and complex geometric descriptors of vessels without potential ambiguities resulting from erroneously detected vessel boundaries. The potential descriptors include, but are not limited to, the central line of the vessel (the skeleton), the diameters of the vessels at each point of the skeleton, volume, tubular area, and more complex descriptors of the vessel shape based on skeletal lines, i.e., tortuosity index, deviation index, and directional cosines of connected vascular segments [17].

Our proposed tubular reconstruction framework delivers several unique capabilities. It allows constraining volumetric trees to reduce areas of interest and final model complexity significantly. Skeletal optimizations proposed in this work apart from classical smoothing allow for user-dependent model complexity simplification. The final stage of tubular reconstruction introduces a new n-furcation model. Other stages of the data processing pipeline implemented in our framework deliver minor customizations and improvements also mentioned in this paper.

We apply the method for the evaluation of the physiological variance of brain arteries detected on time-of-flight magnetic resonance imaging on three Tesla scanner in healthy volunteers. We also propose a systematic approach for the treatment of the tube overlapping problem by detecting the uncertain volume and defining it as a joint object. Our main goal is to develop a semi-automatic and fully interactive method. We introduced a significant number of parameters to define a scenario based on data modality, resolution, image properties, and texture features. We can prepare and fix a set of parameters to apply it for a database representing the same measurement scenario.

## Methods

The proposed approach for the reconstruction of the cerebrovascular system performs several optimization steps on candidates for the initial vessels segmented from the raw MRI data. The goal is to obtain quantitative information on vessel orientation and radius and to assure the continuity and smoothness of the reconstructed network at $n$-furcations of the vascular system. The initial segmentation of the vessels is obtained with the morphological growing by exploiting the intensity remapping for the increased robustness and the insensitivity to intensity relationship between the desired and the unwanted voxels. Thereafter, the result is skeletonized; the obtained skeleton is smoothed, and the junctions of the skeleton are optimized. In the subsequent step, the tubular mesh is applied around the skeleton to encompass the segmented voxels. The collisions inside the tubes are removed with both intratubular collisions and collisions at the n-furcations of the tubes. In the last step, the mesh at the n-furcations is generated to link the tubes. The overview of the algorithm is summed up in Algorithm 1.

Algorithm 1. Enhanced tubular system reconstruction
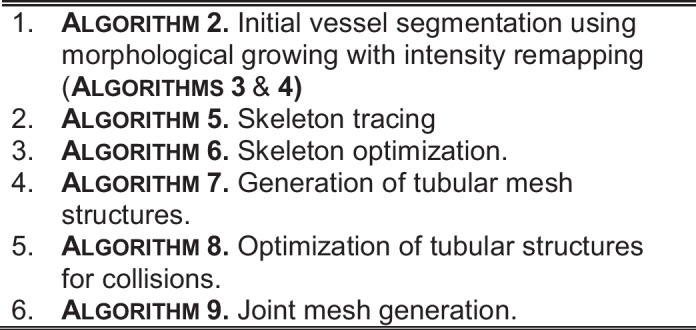


### Morphological growing with intensity remapping

In the initial step of the algorithm, an initial segmentation volume seed $V$ is placed manually by the user. When dealing with pathological cases or discontinuities in segmentation, the method can operate on multiple seed points. Thereafter, in an iterative manner, the neighboring similar pixels are incorporated into the initial volume as shown in Algorithm 2. In our case, the similarity is defined by calculating the following probability proposed by [19].

1 $$P\left( {I\left( i \right)\mid\Phi }\right) = \frac{1}{V(R)}\iiint\limits_{R} {\frac{1}{{\sqrt {2\pi \delta } }}}e^{{\frac{{ - \left( {I(i) - I(r)} \right)^{2} }}{{2\delta^{2} }}}} dr$$

Here, the volume $V$ with $R$ voxels is bounded by the segmentation model $\Phi$. The probability will be maximal when the intensity of the tested $i$-th voxel is equal to the expected value of intensity distribution of the voxels in the volume $V$. In such an approach, one can easily place the same thresholds for segmenting images with different intensity distributions because the same probabilities will be obtained for the voxels with intensity standardized with a standard deviation $\delta$ of the intensity distribution of the voxels $V$. If the probability for a voxel is below a chosen probability threshold, it is rejected; however, if it is otherwise, it is added to the growing volume $V$ and stored for further analysis. The idea is similar to the concept of the shape-based growing model introduced by Masutani et al. [26]. This is implemented using the morphological growth (morphological dilation) manner.

Algorithm 2. Vessel dilation using morphological growing
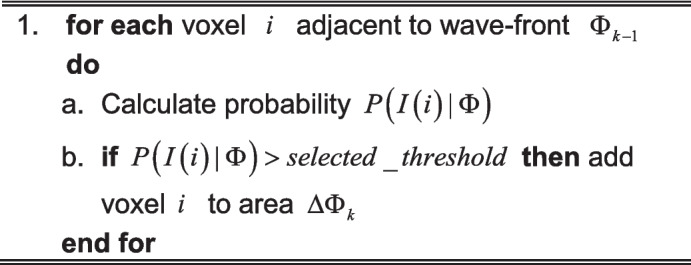


In one $k$-th iteration, all the voxels $\Delta \Phi_{k}$ adjacent to $\Phi_{k - 1}$ are tested using (1). The adjacency is calculated using full cubic $26$ voxels neighborhood kernels. In this approach, the pixels belonging $k$ are known as “wave-front.”

Algorithm 3. Propagation of wave-front in the vessel branch
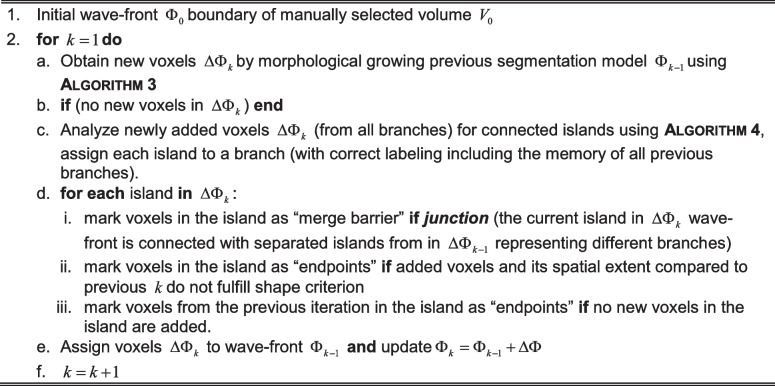


In the described process, the segmentation is growing from the initial estimate and propagates through the anatomical structures of the vascular tree, defined as the graph of connected branches. In each iteration, the voxels that are above the threshold value in the probability given by (1) form a new “wave-front” that is analyzed in terms of connectivity. The aim of this step is to check whether the growing volume enters the new vessels (new branches in the vascular tree), the existing “branches” cease to exist because of a lack of newly assigned voxels, or they are merging with the already assigned voxels. In principle, when the volume enters the vessels, some of the newly added voxels lose connection to the rest of the wavefront, and the wavefront is divided into separated “islands.” Each “island” is the beginning of a new “branch,” given a new unique label, and is propagated until no new voxels are possible to add, or a given “island” touches voxels from a different “branch.” This algorithm is shown in Algorithm 3.

In every iteration of the growing algorithm, we fully control the properties of the growing branches. We have information on the volume size and the spatial extent of the current addition to a given branch. This gives the method the possibility to control the kind of growth that is allowed. By setting the boundary condition for specific geometric properties for newly added voxels, we can block the detection of vascular structures of a certain thickness, or we can block the leakage of region-growing segmentation through small holes.

**Fig. 1:**
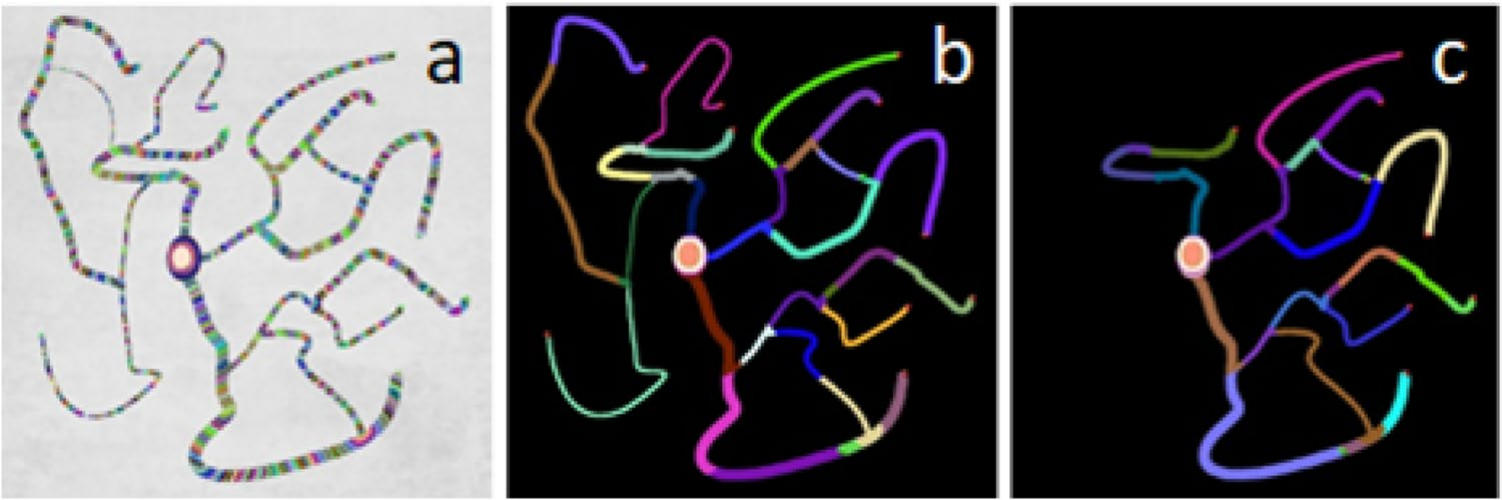
Results of the growing algorithm. **a** Advancing wave-front values marked inside the obtained final segmentation. **b**–**c** Final segmentations with colored branches and boundary conditions on minimal artery diameters (**b**) $5mm$, (**c**) $7mm$

This property is an essential feature of our segmentation method, making it more versatile. Figure 1 presents a 2D version of the implemented algorithm. Wave-fronts $\Delta \Phi_{k}$ from all the iterations are presented with distinct colors in Fig. 1a. Figure 1b and c show the boundary condition effects applied to minimum diameters of new branches with $5mm$ and $7mm$. Several vessels are not detected as the algorithm checks the diameter of every island and stops the propagation of the branch if the diameter of the island is below the threshold. For all labeled branches, we keep the information on its parent branches to be used later in the skeleton tracing process. There are many methods for dealing with segmentation leakage through small holes. The simplest solutions would involve image Gaussian smoothing to soften image gradients or applying grayscale morphology to fill holes by surrounding edge values. Our work extends the holes detection task by controlling the wave propagation properties and their variability. Keeping the wave propagation historical features like its spatial extent or simple volume can be treated for automatic detection of stenosis. Such elements form a data sequence that is a profile of vessel thickness variability.

**Fig. 2:**
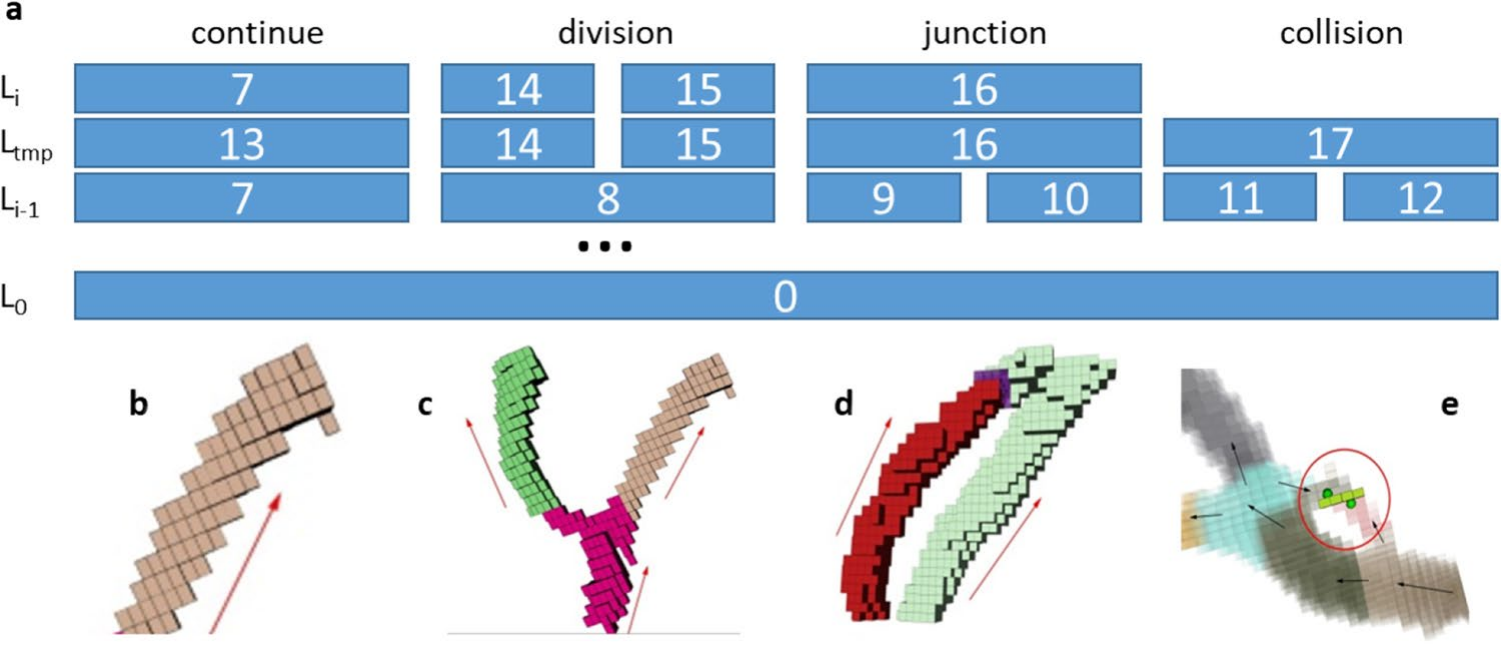
**a** Illustration of the labeling procedure. **b** If one new island connects to one island from a previous iteration, the label propagated as two islands are part of the same branch (vessel). **c** If several new islands emerge from one island from a previous iteration, new branches are created (one vessel divides into more new vessels). **d** If one island links to several islands from previous iterations, one new branch is created (a number of vessels merge into one). **e** If branches collide and no propagation is possible, the algorithm stops

The essential moment of processing the wave-front $\Delta \Phi$ in each iteration is an analysis of the possible division or junction of the separated islands and their assignment to proper branches. This part of the algorithm is presented in Algorithm 4. with an example presented in Fig. 2a. Figure 2b–e presents the visualization of branch continuation in Fig. 2b; the branch division is in Fig. 2c, and the branch junctions are in Fig. 2d–e with arrows representing the direction of the wave-front propagation.

Algorithm 4. Analysis of islands’ connectivity
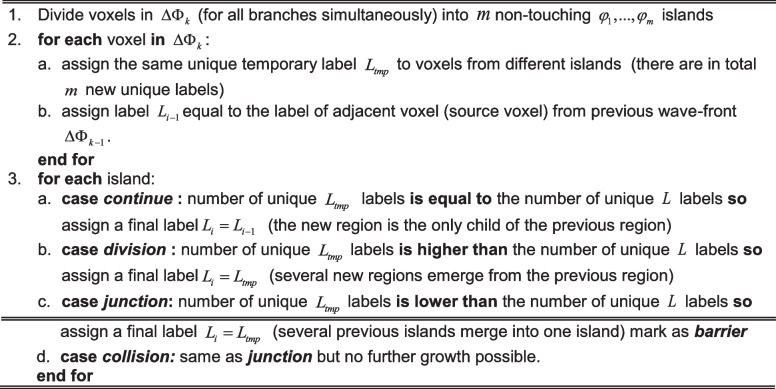


Our skeletonization procedure (described in detail later) requires that the segmented and labeled vascular structures are formed as a tree with no loops. In order to achieve this, we need to apply a procedure to remove the connection between the colliding wave-fronts.

There are two main cases we need to consider to detect and mark junction barriers:

- when two or more wavefronts merge and further propagation is possible, as shown in Fig. 2d,
- when merged wavefronts have no further continuation, as demonstrated in Fig. 2e.

Figure 2e presents the most common case—all voxels detected and marked as junctions are marked as barriers that will block the skeleton tracing. The first case can be more complex. In general, when $m$ wave-fronts coincide and they have to be merged into a new branch, the group with the largest number of voxels (the thickest one) is propagated, and the rest ($m - 1$ groups of voxels of a current growth) is labeled as barriers. Such a solution transforms the graph into a tree with no loops. It has to be emphasized, however, that the information on the colliding branches is preserved, and the vascular loops can be accessed during the vascular network analysis.

**Fig. 3:**
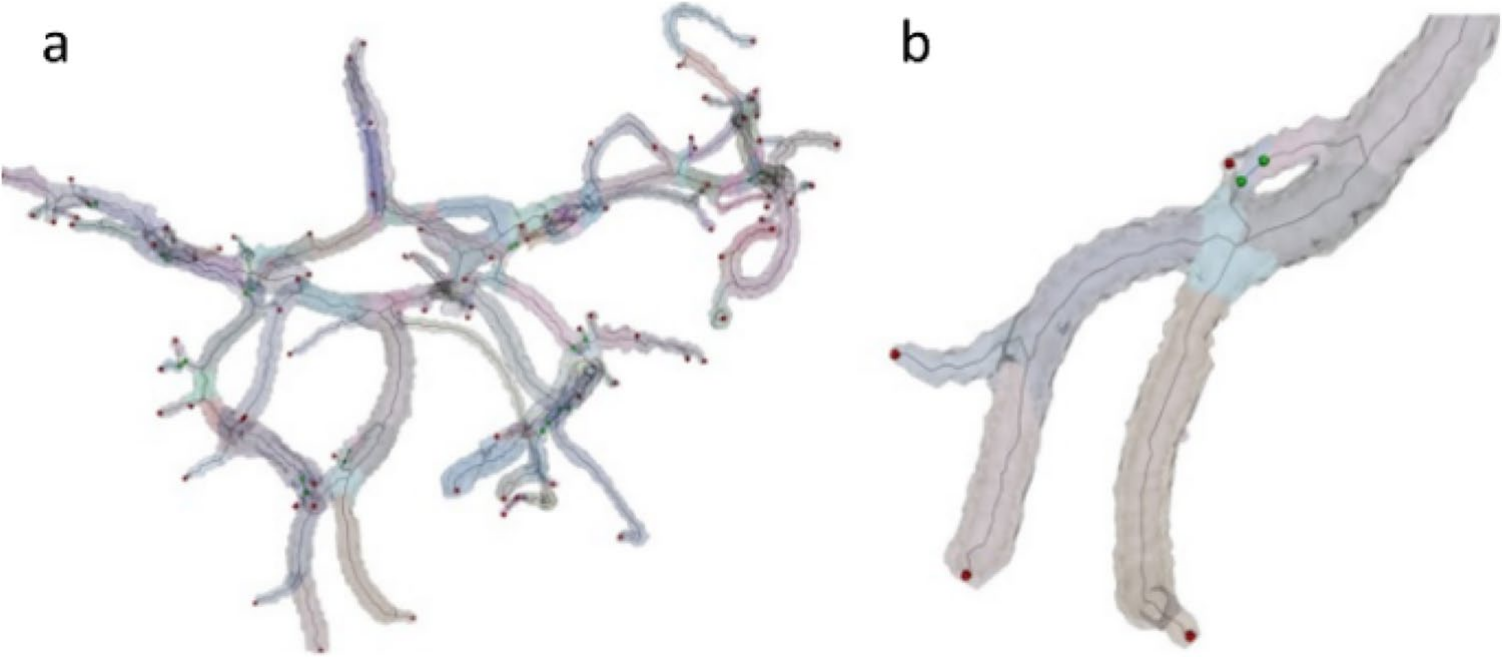
Initial skeleton for data from Fig. 3 traced from wave-front endpoints (red spheres) and branch merging points (green spheres)

As the final result of this step, we obtain a labeled geometrical voxel tree in 3D as shown in Fig. 3a, and the “merge barriers” and “endpoints” in Fig. 3b are the input points for the subsequent skeletonization procedure. Additionally, each voxel has two more values assigned: Euclidean distance [13] from the boundaries and the propagation distance from the seed voxels. The Euclidean distance maps were generated using the algorithm described by [23].

### Skeletonization

Skeletonization can be defined as a process of reducing binary volumetric objects into a curve skeleton with the preservation of its form and topology. It allows a synthetic representation of three-dimensional objects that can be used for simplified shape descriptions based on the extracted centerline and the geometric property analysis. Using skeletonization for vascular, reconstruction is an essential process that needs to be automated. Skelton extraction, when combined with local radius information, allows a synthetic description of vascular details and the detection of possible pathologies. The object centerline extraction methods can be divided into two main groups:

- approaches that are based on thinning volumetric objects using an iterative process of removing external voxels.
- methods that search for the optimal path between selected points.

A wide group of thinning algorithms consists of sequential detection and voxel removal one by one [2, 30]. This approach has a major disadvantage; they rely on an arbitrary choice of voxel removal, and in some cases, it can stop without obtaining the final result. The other strategy proposed in the literature uses distance transform for consequent erosions [2, 9]. Reference [21] used fuzzy distance transform for increasing the efficiency of an algorithm. The second group that searches for the optimal path between points has been widely described in the literature [5, 16, 40]. The optimality criterion can be derived from the image factors (intensities, gradient, and distance from the boundaries). Such methods are often robust in noisy images; nevertheless, they require an initial selection of source and endpoints. Our study has chosen to implement the skeletonization method using the optimal path searching with manual selection of the starting points, using the maximized Euclidean distance from the segmentation boundaries as the cost function in the optimization procedure. We are following the propagating wave-front back-tracing paradigm proposed by [11] for 2D images and [10] for the 3D extension.

**Fig. 4:**
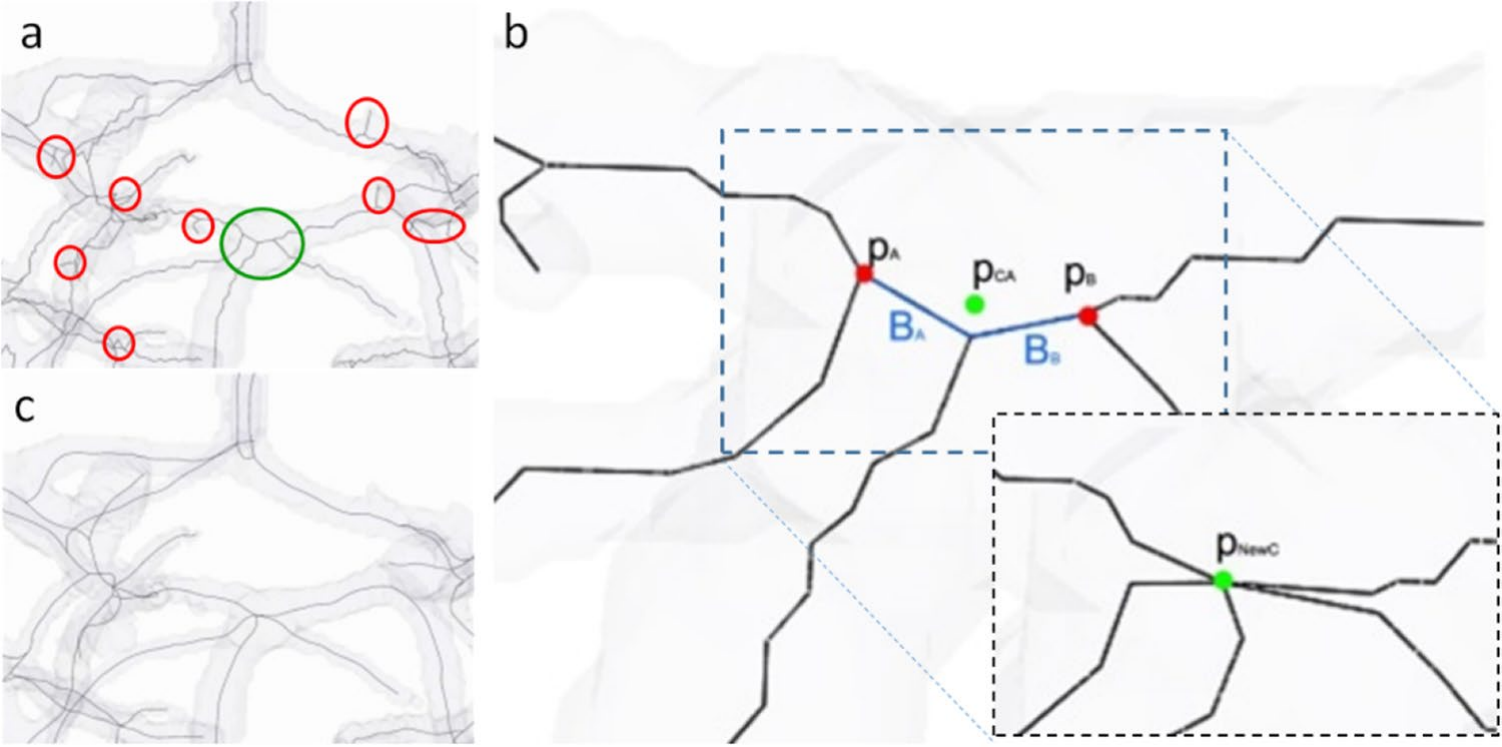
Skeleton optimization phases. **a** Starting skeleton estimation. The red ellipses show regions to be optimized. The green ellipse marks the region presented in b. **b** Joint points optimization—short branch removal and merging. Inset shows joint point after optimization

Our skeletonization procedure described in Algorithm 5 assumes that our processed object of the connected vascular structures is a tree without loops. It performs back-tracing from the red sphere-marked endpoints, followed by tracing from the wave-front-break points marked as green spheres. The following quantities are used in tracing the process for the analyzed voxel $v$: $ED(v)$—Euclidean distance from the segmentation boundary; $ADV(v)$—propagation distance from the seed points; $L(v)$—branch label; $LP_{k} (v)$— $k$-th parent label of a current branch. More than one of the quantities is possible when a branch is created from the junction. An example result of the skeleton tracing process is presented in Fig. 4.

Algorithm 5. Skeletonization
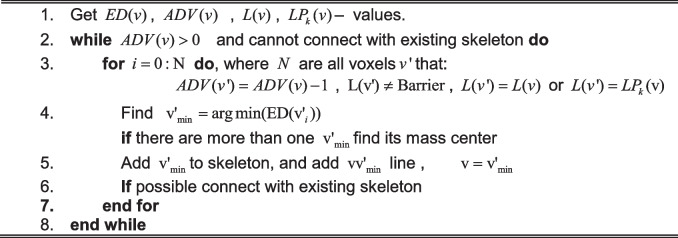


The Euclidean distance maps often have many “flat” regions with identical distance values that may lead to the generation of concurrent parallel skeletons inside the thick vascular structures. Therefore, it is essential to connect the current back-tracing to the existing skeleton where possible. To do so, when we detect the existing skeleton in the close neighborhood of $v$, we check if it can be connected with a straight line to $\mathrm{v}$ $v$ without crossing the segmentation boundary. Such a method can ensure the simplification of the resulting skeleton. The last part of skeleton tracing is the classification of the skeletal points. Every point is analyzed in terms of the number of connections: 1—terminal point, 2—branch interior, 3 or more—junction. We separate every branch, store it as a tree structure, and pass it to the optimization routines. An example of a skeleton after this phase of skeletonization is shown in Fig. 4a.

### Skeleton optimization

In order to simplify the skeleton, the first phase of skeleton optimization involves the removal of short leaves of the defined tree. Short skeletal parts with user-defined tolerance with no connection to one of the endpoints are deleted (red circles in Fig. 4a). The goal of the second phase is to connect the short inner branches that usually occur near joint points where more than three vascular structures are connected, as marked with a green ellipse in Fig. 4a. The inner edge threshold value for minimal length can be selected manually or automatically selected as the diameter of the thinnest vascular structure detected in the reconstructed tree. In the procedure depicted in Fig. 4b, the short branches $B_{0} \ldots B_{k}$ are removed, and their endpoints are merged into a new central point as marked in the subset of Fig. 4b. This new junction point $p_{CA}$ is calculated as the center of mass of the removed segments’ endpoints (points $p_{CA}$ and $p_{CB}$). In the final step, $p_{CA}$ is repositioned into a new position $p_{NewC}$ from the closest neighborhood of $p_{CA}$, maximizing the distance to the segmentation boundary. This last repositioning step can be treated as fine-tuning as we want to keep joint centers as close to the center of segmentation as possible. Such a solution significantly reduces the skeletal model’s complexity and simplifies the final geometrical model. The last part involves optimizing the nodes’ location to obtain smooth curves, as seen in Fig. 4c. It is a standard solution to smoothen the skeleton, and it is mainly done by spline-fitting into the point set defining skeletal segments [46].

Algorithm 6. Skeleton optimization
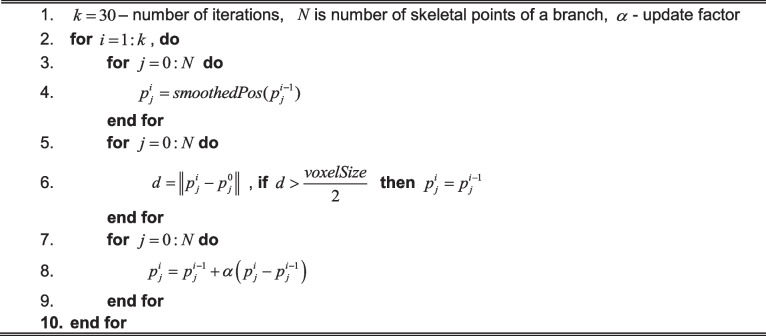


Our study uses a similar method for smoothing as presented in [42]. Algorithm 6 has extended the method for constraining the distance from the boundary surface values that should stay in the selected tolerance. First, we double the resolution of the skeleton using B-Splines interpolation. In order to improve its shape, we apply iterative Gaussian-weighed smoothing of all the skeletal segments separately; we do not reposition the junction points. We use simple convolution formula for the $1D$ smoothing.

$$smoothedPos(x_{i} ) = \frac{{\sum\limits_{j = 1}^{n} {\left( {w_{j} \cdot x_{i} - \frac{n - 1}{2} + j} \right)} }}{{\sum\limits_{j = 1}^{n} {w_{j} } }},$$

where $n$ is the odd size of the weighting kernel, and $w_{j}$ is the weight of the Gaussian kernel. We also do not allow repositioning of the skeletal points further than half of the voxel’s size in all the directions to prevent the optimal distance from bounding the segmentation.

### Primary generation of tubular structures

Our vascular segment of the reconstruction method consists of four phases. In the first phase, we reconstruct the tubular object for every segment. In the second phase, we detect the collisions between the segments and remove the intersecting parts of every segment. Thereafter, we detect and remove the interior mesh collisions. The last part of the reconstruction procedure consists of a mesh generation at every $n$-furcation point. The following subsections contain a detailed description of the developed algorithms.

There are two main models of the tubular structure reconstruction described in the literature. The first one assumes that cross-sections of such objects perpendicular to the skeleton are circles, while the second one claims that the shape of the ellipse is more accurate [31]. In our study, we have selected the circular model because it is less complicated and easier for further optimization. First, we need to estimate the values of the radius along the skeletal segment. Reference [44] used a ray casting technique to trace the gradient maxima from a central point of the vessel in a $2D$ cross-section image. We use a similar approach to determine the radius and use ray casting, taking into account the 3D orientation of the skeletal line. For every point, we cast rays in $N_{dest}$ directions in a plane orthogonal to the polyline, where $N_{dest}$ is the desired density of the final triangle mesh. When we collect the $r_{0} \ldots r_{dest - 1}$ values, we select a median value to filter the out-noise. When we collect an array of the median radii along the skeletal segment, we apply the Gaussian-weighed radius filtering to generate smoother tubular objects.

### Internal collision detection

**Fig. 5:**
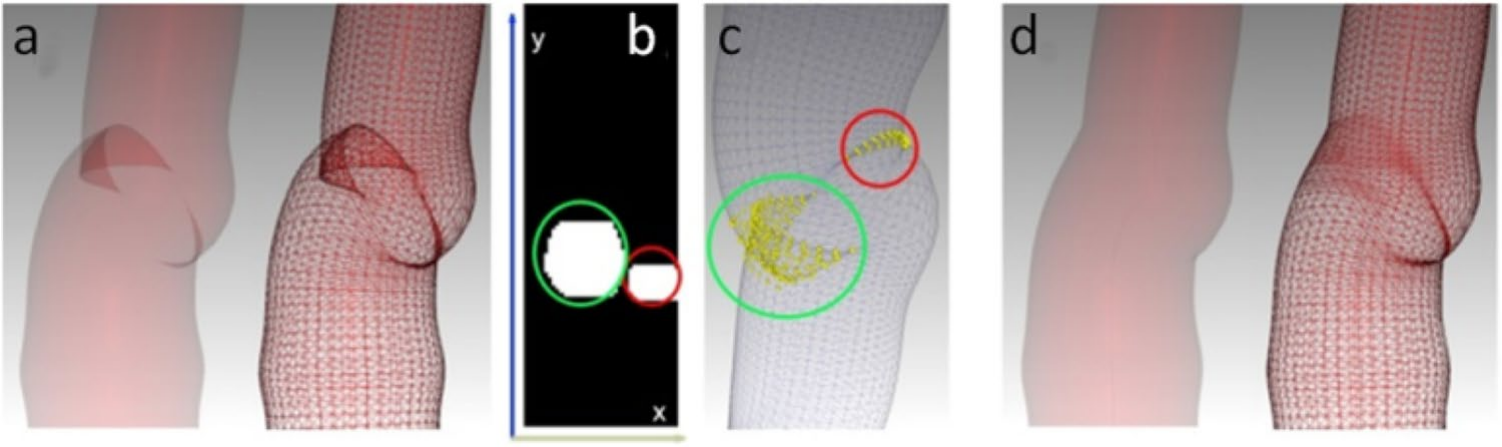
Self-collision removal scheme. **a** Visualization of the colliding mesh. **b** $2D$ array with colliding points detected (in red and green circles). **c** $3D$ visualization of the colliding points (yellow spheres). **d** Visualization of the mesh with collisions removed

Most commercial applications utilize tubular filters that produce triangular meshes connecting discs that are perpendicular to the skeletal line. They are mainly used for determining planes for generating 2D volumetric cross-sections used for the presentation of intensity values along the reconstructed tubular objects. One problem may occur when the curvature is significant for higher radius values. The discs perpendicular to the skeleton may start to collide, as shown in Fig. 5a. This produces self-collisions in the generated mesh. These self-collisions have to be detected and removed. Reference [45] proposed a method of merging the vertices to eliminate the crisscrossing of the radial lines. The second problem that arises is that when we generate the $2D$ planar reconstructions while tracing the skeleton, we can produce false reconstructions that will present anatomical features as being misplaced—when one of the discs that are crossing one part of the colliding disc is in the wave-front and the second is at the back of the second disc. In order to remove such collisions, we need to detect them accurately.

In our study, we use a two-pass algorithm that analyses the points on $DISC_{k + 1}$ lying on one side of $DISC_{k}$, and in the second—backward pass, we analyze every $DISC_{k}$ for collision with $DISC_{k - 1}$. In every step, for a given $DISC_{k + 1}$, we define the Cartesian equation of a plane $a \cdot x + b \cdot y + c \cdot z + d = 0$, where $\left\langle {a,b,c} \right\rangle$ is the vector normal to the plane. Thereafter, we check the analyzed neighbor disc if it is situated at the same side of the plane. When they are not, these points are marked for removal. We create a flag array $dest \times N$ where $N$ is the number of skeletal points. In Fig. 5b, the white pixels are marked as the colliding ones. They are also visualized as yellow spheres on the mesh, as shown in Fig. 5c. After the detection of the colliding points, we apply dilation on the array to remove the points adjacent to the ones from the detected regions. Thereafter, the removed points are interpolated using Catmul-Rom splines [12] in the form of:

$$P(t) = \left[ {\begin{array}{*{20}c} 1 & t & {t^{2} } & {t^{3} } \\ \end{array} } \right]M\left[ {\begin{array}{*{20}c} {P_{i - 1} } \\ {P_{i} } \\ {P_{i + 1} } \\ {P_{i + 2} } \\ \end{array} } \right],{\kern 1pt} \,\;{\text{where }}M = \frac{1}{2}\left[ {\begin{array}{*{20}c} 0 & 2 & 0 & 0 \\ { - 1} & 0 & 1 & 0 \\ 2 & { - 5} & 4 & { - 1} \\ { - 3} & 3 & { - 3} & 1 \\ \end{array} } \right],$$

where $t$ signifies the portion of the distance between the two nearest control points, and $P_{0} \ldots P_{N}$ are the mesh points along the skeleton ($Y$ axis) for a given position at the $X$-axis.

### Tube intersections

**Fig. 6:**
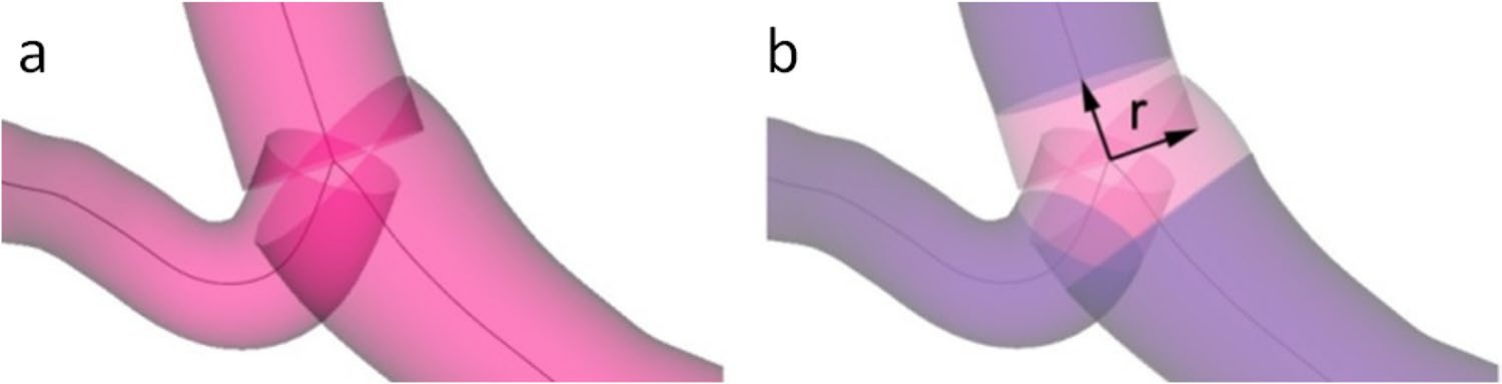
Primary tube clipping using radius checks at the tube ends. **a** The initial state of the fully colliding meshes at the joint point. **b** Tube meshes after the first shortening by the radius estimated for the first skeletal point

Determining the geometrical identification of $n$-furcation is not only a task of centerline analysis. It is also necessary to characterize the $n$-furcation points with reference to reconstruct tubular mesh surfaces. Authors [33] proposed the identification of the bifurcation region of the intersecting tubes by defining the bifurcation plane, detecting the skeleton-surface intersection points as the centers of the maximal inscribed spheres associated with the centerline locations, and splitting the final mesh junction reconstruction between the tubular segments. In this method, the authors did not exclude any volume for the joint object; however, they divided the junction contribution between the colliding tubes. In our method, we use the detection of the collision using voxelized mesh collision with the exclusion of the intersection region, which is a new object referred to as the “joint point.” This volumetric part where the uncertainty of the membership occurs can be separated. The main reason for this uncertainty is that in the neighborhood of the joint point, there are usually many voxels of the same distance value—there can be a “flat” region where many points meet the criterion of a joint point. The size and shape of the dilation 3D kernel can result in slightly repositioned joint points. Such repositioning may result in changes in the skeleton’s directions. Therefore, we have decided to remove the tubes’ collision visible in Fig. 6a by removing the uncertain volume from all of the touching tubes. We apply a tube clipping algorithm to remove overlapping segments, as shown in Fig. 6b.

### Joint reconstruction

**Fig. 7:**
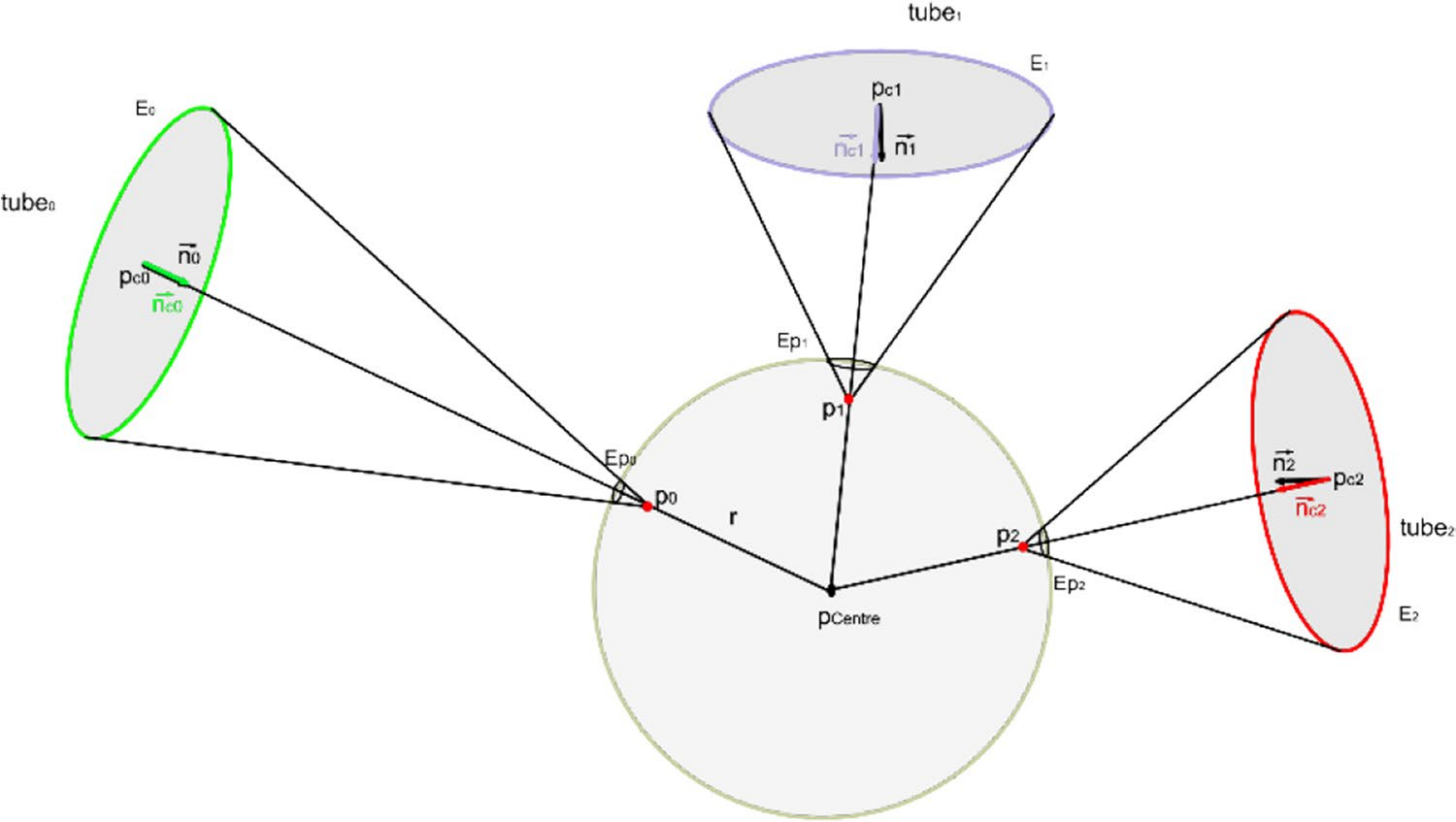
The idea of sphere projection. Three elliptical tube ends are projected onto a sphere. The center of each ellipse is connected to the center of the sphere. The center of each projection is placed on a resulting line and is moved towards the center of the sphere by $\Delta r$ from the collision points of the line and the sphere. All the $dest$ points of the tube ends’ ellipses are projected onto a sphere using this point

The novel joint reconstruction algorithm Algorithm 7 consists of several phases. First, we need to estimate the center $p_{Centre}$ of a sphere and its radius. The sphere must be visible from all ends of the tubes shortened in the previous step. The shape of the ends is in the form of ellipses with the vectors $\overrightarrow {{n_{x} }}$ being normal to the surface of the ellipse facing the sphere as shown in Fig. 7. The final central point is localized at the optimally visible spatial position, and it is estimated using the optimization scheme, minimizing the maximum $\theta_{{k_{i} }}$ angle.

$$p_{{{\text{Centre}}}} = p_{{{\text{i}}_{{{\text{MIN}}}} }} ,{\text{ where }}i_{{{\text{MIN}}}} = \arg \min_{{{\text{i\^I }}\left\langle {\text{0,N - 1}} \right\rangle }} (max(\theta_{{{\text{k}}_{{\text{i}}} }} ),$$

where $\mathrm{N}$ $N$ are all possible skeletal points between the endpoints of the tubes within the joint and

$$\overrightarrow {{c_{{k_{i} }} }} = \left( {p_{{Centre_{i} }} ,p_{{c_{k} }} } \right),\overrightarrow {{n_{{{\text{c}}_{{\text{k}}} }} }} = \frac{{\overrightarrow {{c_{{{\text{k}}_{{\text{i}}} }} }} }}{{\left\| {\overrightarrow {{c_{{{\text{k}}_{{\text{i}}} }} }} } \right\|}},\theta_{{k_{i} }} = \cos^{ - 1} (\overrightarrow {{n_{{c_{k} }} }} \cdot \overrightarrow {{n_{k} }} ).$$

The second part is the ellipse $E_{k}$ projection onto a sphere to obtain a set of $Ep_{k}$ ellipses. For every $k$-th ellipse, the center of the projection is localized on the $\left( {p_{{Centre_{i} }} ,p_{{c_{k} }} } \right)$ line at a distance $r - \Delta r$ from $p_{Centre}$ to $p_{k} = p_{Centre} - \overrightarrow {{n_{{c_{k} }} }} \cdot (r - \Delta r)$, where $r$ is the radius of the projected sphere.

**Fig. 8:**
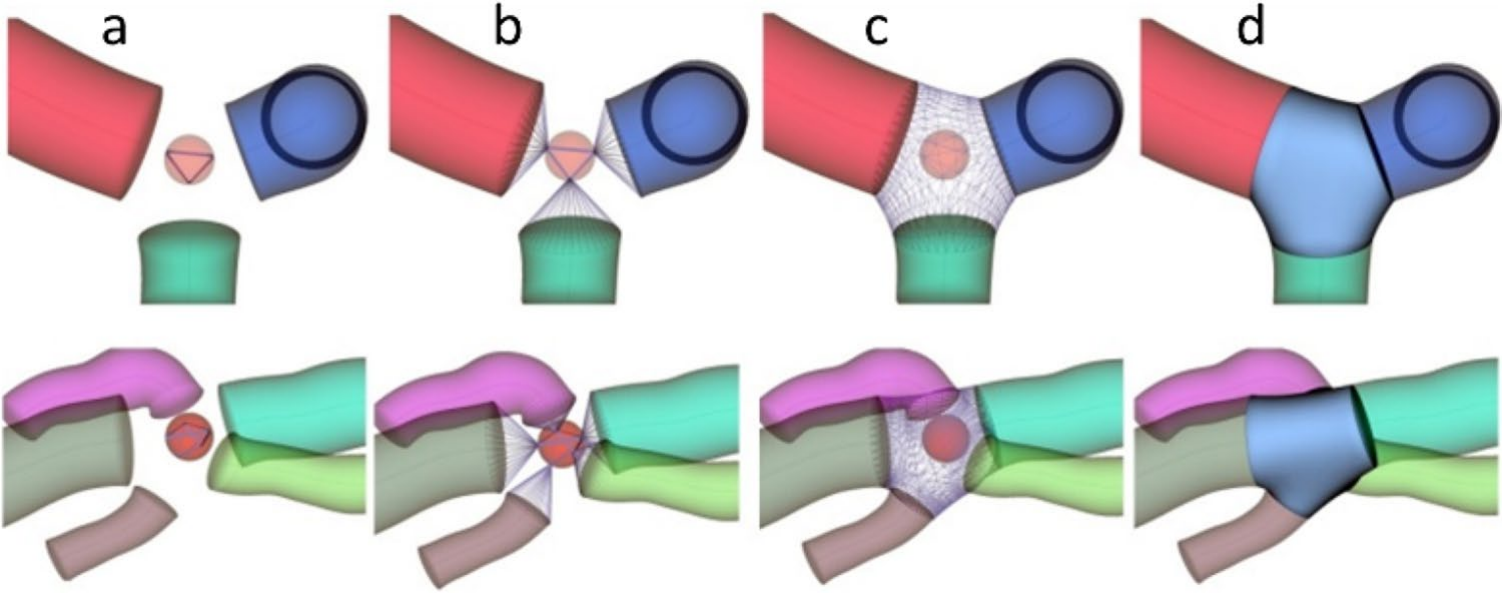
Joint generation algorithm ($r = 0.7 \cdot r_{\max }$) for bifurcation (top row) and quadri-furcation (bottom row). **a** The optimal sphere position in the space between the tube ends with projected circles and Delaunay triangulation. **b** Connection of the Delaunay triangulation to the existing tube endpoints. **c** Result of mesh relaxation. **d** Final visualization of the reconstructed joint

The goal of repositioning the projection center from $p_{Centre}$ to $p_{k}$ is to generate the smallest and most well-separated projected ellipses (with maximized distance from other projected ellipses) to avoid collisions. The next step is to generate the Delaunay triangulation [14] of the projected ellipses. Such a triangulation can be applied straightforwardly to generate the connection between projected ellipses, resulting in a convex hull, as shown in Fig. 8a. Thereafter, the new triangulation can be connected to the original tube’s ends, as can be observed in Fig. 8b by linking the corresponding vertices. The last step, as shown in Fig. 8c–d, involves mesh relaxation using constrained Laplacian smoothing [15, 29]. The selection of $r$ and $\Delta r$ may be crucial for the smoothing process. In order to avoid large initial angles between the Delaunay triangulation and the mesh connecting it to the tube ends, we experimentally selected $0.7$ as the maximum possible $r$ value $r_{\max }$ (distance between ${\mathrm{p}}_{\mathrm{Centre}}$ $p_{Centre}$ and the closest point of all the tube ends ellipses) and an example set of the joint reconstruction with $r = 0.7 \cdot r_{\max }$. In our experiments, we are relying on the Laplacian mesh smoothing constrained smoothing implemented in the VTK [36] library. Therefore, it will not be discussed in this study.

Algorithm 7. Joints generation
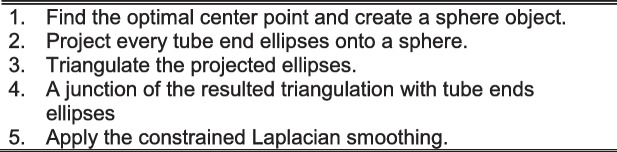


The resulting mesh has excellent properties for further mesh modifications and the generation of the volumetric meshes for simulations—no collisions, no elongated triangles, and no triangles degenerated to a line.

## Results

A sample of $20$ $3D$ time-of-flight magnetic resonance angiography images acquired on three Tesla scanner (Philips Intera Achieva, Nijmegen, Netherlands) at the London Hammersmith hospital was used to evaluate the algorithm. The data is publicly available on the IXI dataset website (http://brain-development.org/ixi-dataset/). The parameters of each sequence are as follows:

$$TR \, = \, 16.7 \, ms;TE \, = \, 5.7 \, ms;phase \, encoding \, steps = 286,$$

$$ETL \, = \, 0;acquisition \, matrix \, = \, 288 \, x \, 286;$$

$$reconstruction \, diameter \, = \, 240.0;the \, flip \, angle \, = \, 16.0.$$

The voxel size of the analyzed datasets was equal to $0.47 \, mm \times 0.47 \, mm \times 0.8 \, mm$.

For each dataset, the same procedure was performed. The operator manually indicated the starting point for the morphological growing inside the basilar artery and obtained the labeled voxels of the initial vessel tree segmentation and the simplified skeleton. At this stage, the branches of the vessel tree could be removed if, for example, only the selected parts were of interest. Thereafter, the optimized skeleton and the tubular model with the joints were created automatically. Depending on the complexity of the vessel tree in a given dataset, the total calculation time is below 10 s on a personal computer with Intel I7 4702QM (3.2 GHz, 4 cores/8 threads), 16 GB of RAM, and NVidia GTX 765 M.

First, we checked the repeatability of the morphological growing and its robustness to the initial choice of seed. We launched the morphological growing procedure $50$ times, starting with seed volumes randomly selected from the basilar artery. In each case, the whole procedure consisted of a morphological growing, initial and optimized skeleton formation, and finished with the tubular reconstruction of the vessel branches.

**Fig. 9:**
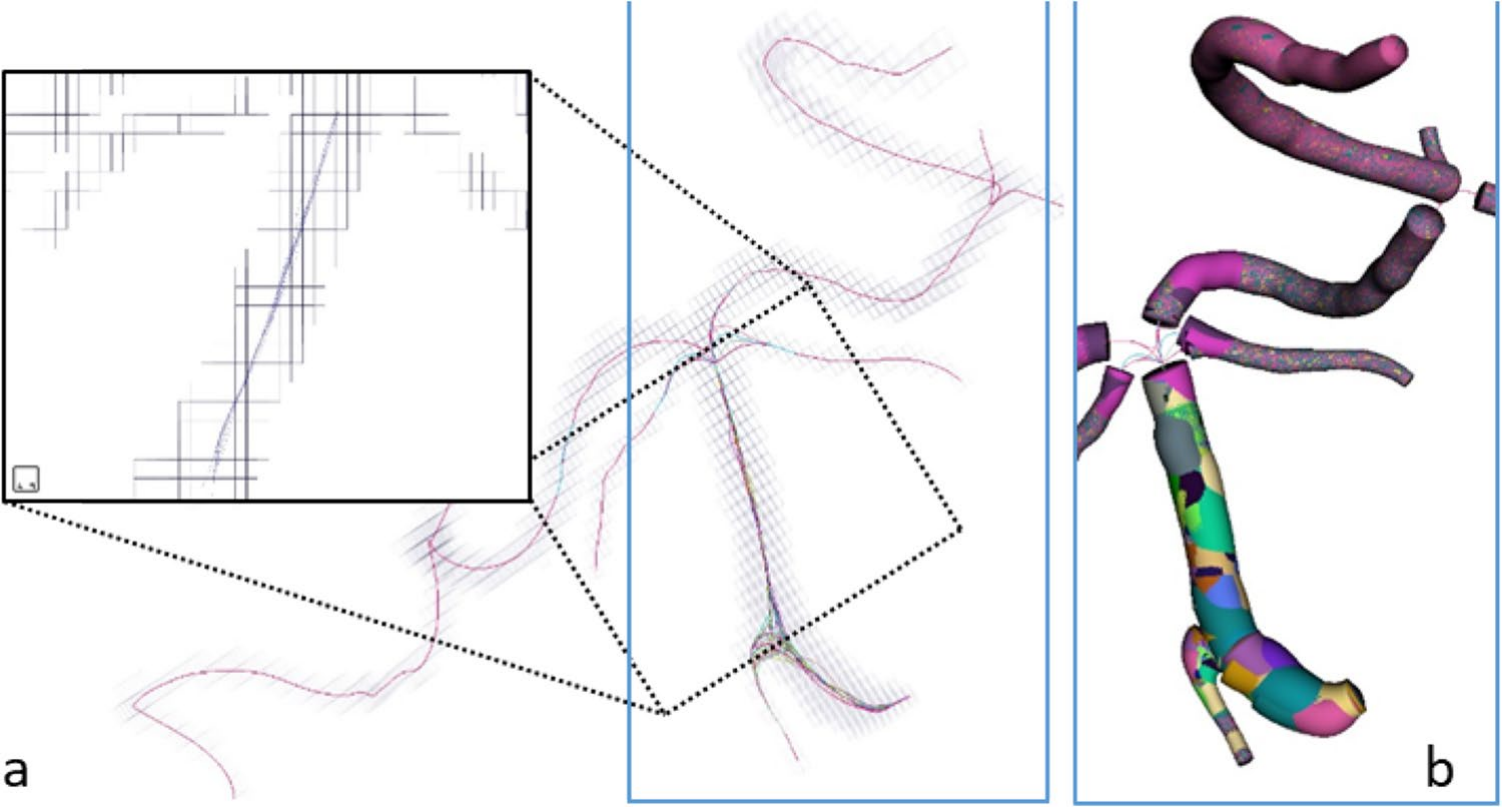
Robustness of the method to the choice of the initial seed in the morphological growing in the sample case from the IXI database (606–2601). **a** Fifty skeleton lines (purple, smoot lines inside) overlaid on the voxels from the morphological growing (perpendicular lines mark the edges of voxels). **b** Tubular segments are found for each of the skeleton lines. Each of the tubes is rendered with different colors, and only the most external is visible

In Fig. 9a, one can see the skeleton centerlines from all the realizations of the procedure overlaid on the voxels of the data for a fragment of the vascular tree. It can be seen that the differences are almost invisible for the tubular parts of the reconstructed vessels. The higher differences appear only in the proximity of the joints and are later removed in the procedure of joint reconstruction. In order to quantify these higher differences, we calculated the Hausdorff distance [20] that shows the maximal distance between the points from two curves. The average distance between the curves plotted in Fig. 9a is equal to $0.73 mm$, with the maximal value reaching $3.3 mm$. The maximum Hausdorff measure values correspond with cases where the algorithm selected a random seed point inside a joint. As the joint object is not tubular, its centerline can vary—in the Euclidean distance map guiding the search for centerlines—and there are more flat regions with identical maximum values. In other words, the joint represents a region of uncertainty, where neighboring voxels may belong to skeletal lines of different vessels. The solution is to select a seed point outside the joint area. In all cases, a single seed point was enough to reconstruct all the vessels in the vascular tree, but more than one seed may be required in case the parts of the tree are not connected.

The majority of the maximal differences are below the maximal size of the voxel. In Fig. 9b, we show that the tubular segments of the vessels built with the 50 centerlines are practically identical, with the radius below the voxel’s size. This shows that the technique is almost insensitive to the human factor in initializing the segmentation procedure.

**Fig. 10:**
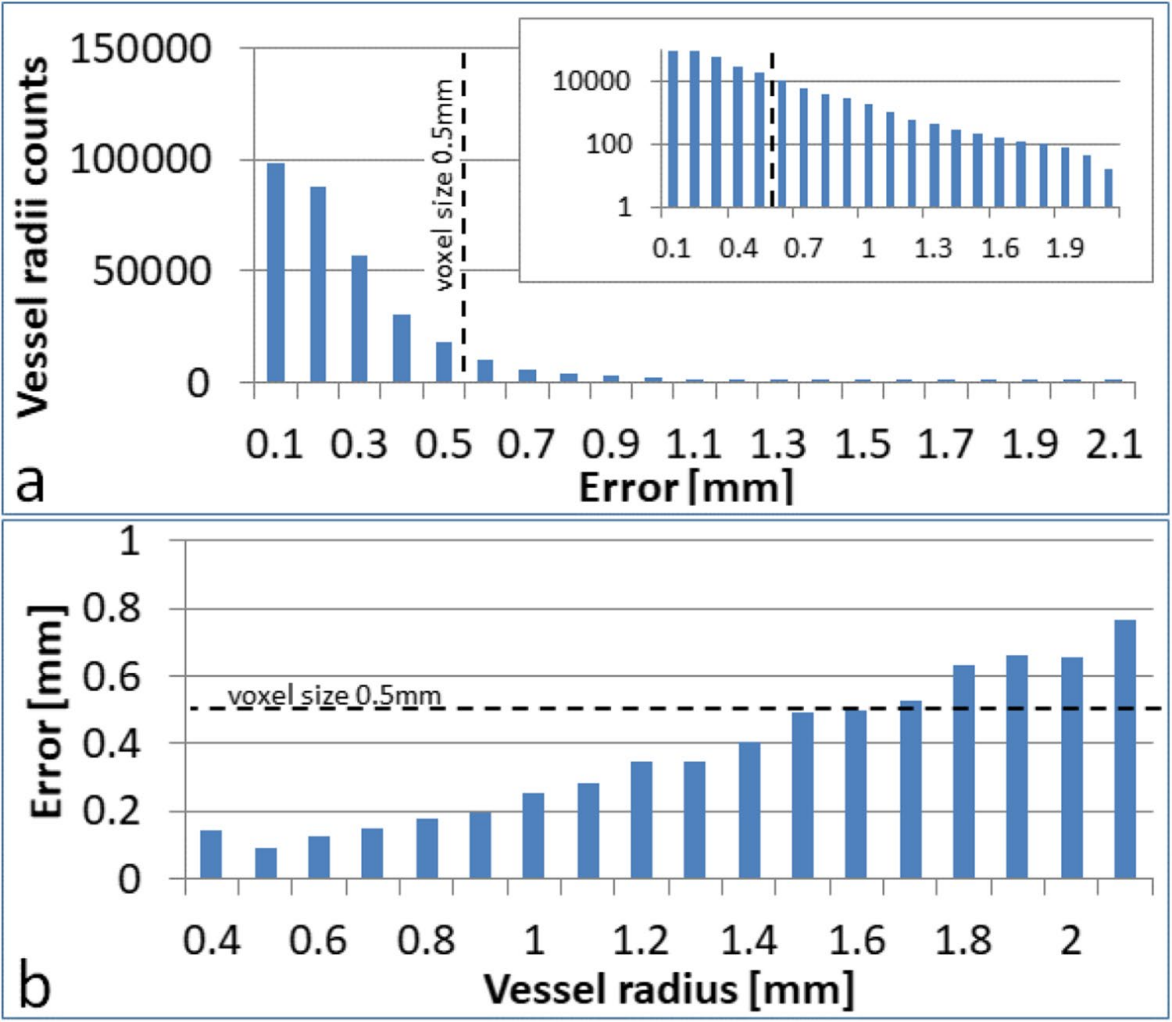
Tubular model reconstruction error. **a** Histogram of the error in the linear and logarithmic scales for all the vessel segments in the selected dataset (603–2701). **b** Mean error as a function of the vessel radius

After that, we checked the accuracy of the tubular approximation of the morphological segmentation. We defined the error of this approximation as the distance of the reconstructed tubular objects from the initial voxel segmentation. This value was calculated for every point of the optimized skeleton for each voxel along the radius of the vessel. The distributions of this error for all of the analyzed datasets can be found in Fig. 10a. An average error for all the cases was equal to $0.22mm$ with a standard deviation equal to $0.20mm$.

For the majority of the tubular radii $\left( {91.4\% } \right)$, the error is lower than the voxel size. Only $0.67\%$ of the errors were larger than $1 mm$, and $0.011\%$ were larger than $2 mm$. In the latter case, the number of points is negligible, and it is only visible in the logarithmic scale in the inset of Fig. 10a. We have also observed that the error tends to increase with the vessel’s radius and reaches maxima for ICA arteries, as shown in Fig. 10b. In such cases, the elliptical model of the vessel cross-section will be more accurate; however, as the number of such erroneous voxels is almost negligible, it is clear that the assumption of a circular cross-section of the tubular segment is sufficient. Additionally, the most significant values above $2 mm$ follow morphological growing properties in regions with large curvature and significant radius values. In the latest implementation, we detect these cases and reposition centerlines towards maximum values in the Euclidean distance map.

**Fig. 11:**
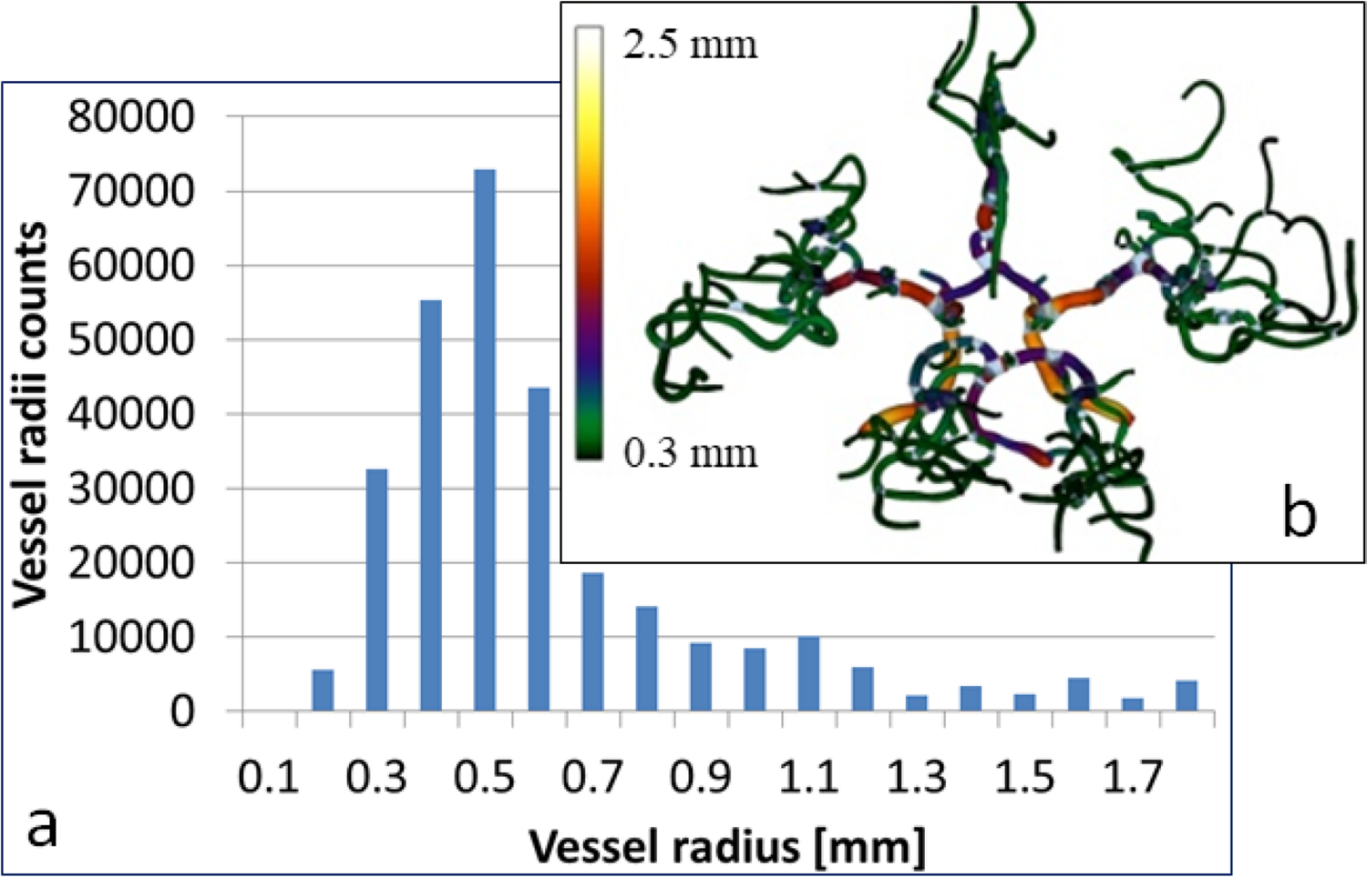
**a** Histogram of the vessel diameters in linear and logarithmic scales for all the vessel segments in one selected dataset (631–2651). **b** The tubular model with the color-coded vessel diameter. Visualization 1 shows the process of morphological growing as well as the consecutive steps of the algorithm overlaid on the cerebral structures

Access to the complete information (such as volume, cross-section area, and skeletal line parameters like length, directional cosines, tortuosity, and other shape descriptors) on every vessel segment enables the quantitative analysis of the vascular structures. An example of simple analysis is shown in Fig. 11a, where the histogram of vessel radii in the whole dataset is presented. The same data is presented in Fig. 11b, where the tubular model with the color-coded vessel diameter is plotted. Subsequently, a more sophisticated analysis is performed for all the $20$ datasets to compare the vasculature of the left and right cerebral hemispheres. A previous study [32] aimed at measuring the lateralization indices of the cerebral flow measurement in humans to evaluate whether the flow acceleration resulted from the vessel stenosis or reduced the distal resistance. The radius asymmetry can impact the flow measurement, and together, they can provide a better understanding of the flow velocity attenuation in the cerebral vessels. To compare the asymmetry for the left and right $M1$ segments, we computed the average radius of the left $M1$ segment ($R_{l}$), the average radius of the right $M1$ segment ($R_{r}$), the average cross area of the left $M1$ segment ($A_{l}$), and the average cross area of the right $M1$ segment ($A_{r}$). Thereafter, we derived the four parameters that we use to quantify the symmetry of the vascular system:

$$\begin{array}{*{20}c} {F_{1} = \frac{{A_{l} }}{{A_{r} }}} & {F_{2} = \frac{{A_{l} - A_{r} }}{{A_{l} + A_{r} }}} & {F_{3} = \frac{{R_{l} }}{{R_{r} }}} & {F_{4} = \frac{{R_{l} - R_{r} }}{{R_{l} + R_{r} }}} \\ \end{array}$$

The results summarized in Table 1 show that the variability of the vessel radius lateralization indices in the sampled individuals is measurable and is characterized by a substantial interquartile range. The radius asymmetry can impact the flow measurement, and together, they can provide a better understanding of the flow velocity attenuation in the cerebral vessels. The structural basis of the flow lateralization is especially critical for understanding the dynamic flow phenomena during cognitive tasks [41] and the development of intracranial atherosclerosis preceding the large vessel ischemic stroke.

**Table 1:** Symmetry analysis of $M1$ segments for $20$ cases from IXI set

Data name	$(R_{l} )$ $[mm^{2} ]$	$A_{l}$ $[mm^{2} ]$	$(R_{l} )$ $[mm^{2} ]$	$A_{r}$ $[mm^{2} ]$	$F_{1}$	$F_{2}$	$F_{3}$	$F_{4}$
603–2701	1.37	5.88	1.33	5.60	1.05	0.02	0.83	− 0.09
605–2598	1.17	4.31	1.29	5.25	0.82	− 0.10	1.46	0.19
606–2601	1.19	4.44	1.20	4.52	0.98	− 0.01	1.27	0.12
608–2599	1.43	6.39	1.40	6.18	1.03	0.02	0.76	− 0.13
609–2600	1.40	6.17	1.12	3.92	1.58	0.22	0.75	− 0.14
610–2649	1.17	4.33	1.01	3.23	1.34	0.15	0.41	− 0.41
611–2650	1.06	3.51	1.08	3.69	0.95	− 0.03	1.00	− 0.00
612–2688	0.98	3.03	1.07	3.59	0.84	− 0.09	1.30	0.13
613–2734	1.26	5.02	1.29	5.23	0.96	− 0.02	0.92	− 0.04
614–2735	1.30	5.31	1.28	5.14	1.03	0.02	1.54	0.21
631–2651	1.38	6.01	1.23	4.74	1.27	0.12	0.94	− 0.03
632–2652	1.30	5.30	1.24	4.84	1.10	0.05	0.63	− 0.23
633–2689	1.24	4.80	1.18	4.37	1.10	0.05	1.23	0.10
634–2690	1.19	4.46	1.21	4.59	0.97	− 0.01	1.49	0.20
635–2691	1.37	5.90	1.32	5.51	1.07	0.03	0.70	− 0.18
636–2733	1.20	4.55	1.11	3.85	1.18	0.08	0.76	− 0.14
637–2785	1.30	5.35	1.28	5.12	1.04	0.02	0.60	− 0.25
638–2786	1.35	5.76	1.28	5.13	1.12	0.06	0.92	− 0.04
643–2787	1.23	4.72	1.24	4.80	0.98	− 0.01	1.64	0.24
646–2653	1.29	5.22	1.29	5.26	0.99	− 0.00	0.92	− 0.04
P10	1.16	4.23	1.08	3.68	0.94	− 0.04	0.63	− 0.23
Q1/P25	1.19	4.46	1.17	4.26	0.98	− 0.01	0.76	− 0.14
Q2/P50	1.28	5.12	1.24	4.82	1.04	0.02	0.92	− 0.04
Q3/P75	1.36	5.79	1.29	5.24	1.11	0.05	1.28	0.12
P90	1.38	6.03	1.32	5.52	1.28	0.12	1.50	0.20
IQR	0.17	1.34	0.13	0.98	0.13	0.06	0.52	0.26
Bowey skew	− 0.03	0.00	− 0.20	− 0.15	0.10	0.04	0.38	0.24
Kelly skew	− 0.04	0.01	− 0.33	− 0.24	0.43	0.30	0.32	0.11

*P*, percentile; *Q*, quartile; *IQR*, interquartile range

The main reason for quantification is the need to describe the cerebral vasculature asymmetry using numbers and aim to compute the asymmetry at a single time-point and follow this parameter’s evolution over time. To identify abnormal asymmetry, one must understand the normal variability of the cerebrovascular tree in healthy individuals [7]. Such variability in vascular asymmetry results from events in the early course of brain development and defines the range where asymmetry is considered within the normal range. Measurement of the cerebral arteries’ lateralization indices will also indicate the potential locations for the flow abnormalities, resulting in subsequent vessel pathology.

Once the asymmetry goes beyond the defined range, one can consider the potential pathological reasons for such deviation. The branching pattern of the cerebral arterial vessels is a complex field that still poses many unanswered questions [18]. We see the following reasons for quantifying the directional asymmetry: clinical and anatomical sciences find structural lateralization of the cerebral structure and function important [28], potential clinical scenarios that might benefit from the fast and automatic computation of those indices rely on qualitative and quantitative evaluation of collateral flow in cerebral vessels. We can provide both the directed and absolute lateralization indices. The impact of automatic vs. manual measurement favors the automatic methods for vessel diameter estimation.

The vessel length and radius asymmetry apply to both ischemic stroke evaluation and arterial vasospasm in the course of the subarachnoid hemorrhage. In both scenarios, the evaluation of flow volume asymmetry influences the diagnostic and therapeutic steps. Therefore, computing the reference range for healthy individuals provides help in the identification of pathological cases. Information on the length and asymmetry of the middle cerebral artery is important and useful in evaluating patients diagnosed with large vessel occlusion and considered for treatment using mechanical thrombectomy, a method recently introduced for the treatment of severe ischemic stroke.

## Discussion

Understanding vascular health in the course of life, development, aging, and disease requires developing new methods of vessel quantification. The aim of this study was to present a vessel segmentation tool for medical professionals that would be easy to use and would provide quantitative data on the vascular tree. The proposed approach to the segmentation of vessels was evaluated using publicly available IXI time-of-flight magnetic resonance angiography datasets. The sequence parameters and hardware settings are representative of the widely used methods applied for clinical evaluation of brain vasculature using pulse sequence that has been available for the last 3 decades.

The unique capabilities of our reconstruction method presented in this study are that it is robust, highly flexible, and resistant to the segmentation errors that arise from human mistakes or lack of experience. In our implementation, we also provided several parameters readily accessible for modification for a user when reconstructing complex vascular geometries from clinical images. Customized constrained vascular volumetric tree generation, skeletal optimizations, and reconstruction of the vascular geometry at n-furcating vessel junctions are the key components described in this work. Therefore, the method is easily to be adapted to many imaging technologies that provide 3D medical data because of the many parameters that can be tuned for optimal results. In this study, we described the application of the method to MRI data; nevertheless, we have started to perform initial tests using CT or OCT data.

The initial segmentation of the vessels starts from the seed points that are selected manually and propagated automatically based on the signal and the spatial properties of the data. In the first phase of the analysis, we obtain the volume of the vascular trees based on the initial selection and signal to noise the properties of vessels. As we have shown, the algorithm is very resistant to the initial selection of the seed; however, during the stage of the procedure, a user can select the shape and size of the voxel adjacency kernel. This is useful in dealing with different data resolutions and allows the rejection of vessels below a certain diameter.

In the second phase, the quantification of the centerlines and the branch terminal points enable more precise identification of the global structure of the brain arteries and the quantification of the properties like cross-sectional area along the individual points belonging to the centerline. Skeleton smoothing provides the opportunity to trim the extracted vascular tree based on the spatial properties anticipated in cerebral arteries. The skeleton can be simplified in many ways by separately treating the short leaves of the skeleton tree, the internal loops (two different nodes having two common branches), and single node loops (both ends of a branch are connected to the same node). The skeletal branches representing the leaves (one free end of a branch) can be shortened when turning towards the boundary. Some endpoints generated in the first step of the algorithm can be manually selected to be ignored in the reconstruction procedures. For large curvatures and radius values, an algorithm in its original form can produce an inaccurate skeleton because of volumetric growth and the splitting process into new volumetric wave-fronts. However, as shown in the results section, the final centerlines in the vessel branches are almost independently identical to the seed selection, and the smooth skeletons provide a convenient visual aid for the evaluation of the major vascular trunks. The only important differences appear close to the vessel joints; nevertheless, these areas are discarded during the later stages of the procedure.

The third step is the reconstruction of the tubular vessel models that are also insensitive to human interaction and lead to almost the same result independently of the initial seed selection. In the final step of the tubular reconstruction method, we can apply a correction procedure to improve the final shape of the tubular segments. For example, the overlap of the vessel lumens enables the identification of the collision discs and identifies the location of the vessel junctions. Trimming of the skeletons based on the location of the collision discs provides a convenient way of locating regions where the vessel geometry is altered by a division in two or more vessels. The final tube and junction model divides the identified vessels into segments and multi-furcations. The segments are characterized by length, width, width variance, and tortuosity. The junctions are defined by the number of inflowing and outflowing vessels, their lumen ratio, and the spatial position.

In our method, we do not highly optimize the vessel joint’s shapes in terms of the distance from the segmentation. Their main role was to fulfill the volumetric mesh continuity criteria. Because of that reason in the error measurement process, the joints were not taken into consideration. The most important was the resection of uncertain volume in the reconstructed tree. Further processing of junction objects would involve expanding to the segmentation boundaries and smoothing. However, it goes beyond the scope of this study, as we are only focusing on tubular structures.

The other problem with the optimized joints is that some reconstruction cases are faulty. The problem arises when the central point of the joint is not visible from all endpoints of the joined tubular segments. In such cases, we should apply a different reconstruction method that is currently developed. This problem can occur when we are setting the skeletal simplification to a high level—many short internal branches are deleted and joined into a single joint for a large number of tubular segments. It is a sporadic case and could occur only when a significant number of tubes would join. In most cases, selecting a proper threshold for the minimum length of the inner segment would remove the problem. In our solution, we can select this parameter manually or we can use the minimal diameter value of the reconstructed tubular tree. The proposed approach also enables the spatial analysis of the vessel lumen and the junction distribution that can be characterized by the field-theory approach to the cerebral vascular tree. The combined dataset of junction locations and their characteristics gives a convenient way to compare the laterality indices of the brain arteries using semi-automatic algorithms for mid-line symmetry detection.

The main advantage of our method presented in this work is that we can adjust to the specific data and case studies. Skeleton reconstruction parameterization gives the user possibility to choose what kind of segments he is interested in and what wants to remove from the reconstruction. The user can select the length, diameter, or angular properties of all analyzed model parts. Separate treatment of leaf branches and inner parts of a skeleton helps in tuning the method for specific datasets and different modalities. Another essential part of our approach is the tube clipping part removing the uncertainty regions from further reconstruction and analysis.

From the point of view of computational complexity, the most critical first-stage segmentation is performed incrementally. The number of steps in this procedure is limited by the size of the *N*^3^ volume, where each voxel is visited at most once, and a fixed complexity procedure is made on it. The remaining parts of the procedure work on a tree, the number of nodes of which is limited by the size of the volume, and the rest of the calculations are linear due to the size of the tree (constant in each node).

We also plan to exploit the versatility of the method to use it with different, more complex vascular geometries, such as tumors or other organ systems [38]. We also plan to explore its application for other data modalities and resolution. Here, one example may be optical coherence tomography that also allows the reconstruction of vascular networks in the human eye [34]. In their studies, the current methodology relies on the ability to identify vessel lumen and probable directions beyond the n-furcation of the vessel. Further steps in the development of such algorithms will greatly benefit from a combination with vessel modeling presented in this study and allow simultaneous calculations of flow direction as well as shape and the diameter of the vessels, and a combination with the analysis of the vessel lumen will allow the assessment of stenosis and vasospasm. This can lead to information on the elasticity of the vessel walls. This study contributes to the development of a generalized vascular model spanning across the meso- and micro-scales that can be used to help in the assessment of the condition of the vascular system in various systemic disorders.

## References

[1] Antiga L, Ene-Iordache B, Remuzzi A (2003). Computational geometry for patient-specific reconstruction and meshing of blood vessels from MR and CT angiography. IEEE Trans Med Imaging.

[2] Arcelli C, Di Baja GS (1985) A width-independent fast thinning algorithm. IEEE Trans Pattern Anal Mach Intell PAMI-7(4):463–474 10.1109/tpami.1985.4767685 21869284

[3] Auricchio F, Conti M, Ferrazzano C, Sgueglia GA (2014). A simple framework to generate 3D patient-specific model of coronary artery bifurcation from single-plane angiographic images. Comput Biol Med.

[4] Aylward S, Pace D, Enquobahrie A, McCormick M, Mullins C, Goodlett C, Reynolds P (2012). TubeTK, segmentation, registration, and analysis of tubular structures in images.

[5] Benmansour F, Cohen LD (2011). Tubular structure segmentation based on minimal path method and anisotropic enhancement. Int J Comput Vision.

[6] Berkhemer OA, Fransen PS, Beumer D, van den Berg LA, Lingsma HF, Yoo AJ, Schonewille WJ, Vos JA, Nederkoorn PJ, Wermer MJ, van Walderveen MA, Staals J, Hofmeijer J, van Oostayen JA, Lycklama a Nijeholt GJ, Boiten J, Brouwer PA, Emmer BJ, de Bruijn SF, van Dijk LC, Kappelle LJ, Lo RH, van Dijk EJ, de Vries J, de Kort PL, van Rooij WJ, van den Berg JS, van Hasselt BA, Aerden LA, Dallinga RJ, Visser MC, Bot JC, Vroomen PC, Eshghi O, Schreuder TH, Heijboer RJ, Keizer K, Tielbeek AV, den Hertog HM, Gerrits DG, van den Berg-Vos RM, Karas GB, Steyerberg EW, Flach HZ, Marquering HA, Sprengers ME, Jenniskens SF, Beenen LF, van den Berg R, Koudstaal PJ, van Zwam WH, Roos YB, van der Lugt A, van Oostenbrugge RJ, Majoie CB, Dippel DW (2015). A randomized trial of intraarterial treatment for acute ischemic stroke. N Engl J Med.

[7] Bishop DV (2013) Cerebral asymmetry and language development: cause, correlate, or consequence? Science 340(6138):1230531 10.1126/science.1230531 PMC4031634 23766329

[8] Bogunović H, Pozo JM, Cárdenes R, San Román L, Frangi AF (2013). Anatomical labeling of the circle of willis using maximum a posteriori probability estimation. IEEE Trans Med Imaging.

[9] Borgefors G (1996). On digital distance transforms in three dimensions. Comput Vis Image Underst.

[10] Cárdenes R, Bogunovic H, Frangi AF (2010) Fast 3D centerline computation for tubular structures by front collapsing and fast marching, 2010 IEEE International Conference on Image Processing. IEEE, pp 4109–4112

[11] Cárdenes R, Ruiz-Alzola J (2005) Skeleton extraction of 2D objects using shock wave-front detection. In: International Conference on Computer Aided Systems Theory. Springer, pp 392–397

[12] Catmull E, Rom R (1974) A class of local interpolating splines. In: Computer aided geometric design. Elsevier, pp 317–326

[13] Danielsson P-E (1980). Euclidean distance mapping. Comput Graphics Image Process.

[14] Delaunay B (1934) Sur la sphere vide. Izv. Akad. Nauk SSSR, Otdelenie Matematicheskii i Estestvennyka Nauk 7:1–2

[15] Field DA (1988). Laplacian smoothing and Delaunay triangulations. Commun Appl Numer Methods.

[16] Flórez-Valencia L, Morales Pinzón A, Richard J-C, Hernandez Hoyos M, Orkisz M (2015) Simultaneous skeletonization and graph description of airway trees in 3D CT images. Proceedings of the 25th GRETSI

[17] Gielecki J, Żurada A, Gajda G, Nowak D, Sienkiewicz-Zawilińska J (2008). The description of vascular variations in three dimensional space: a novel method of spatial cerebral arteries evaluation. Med Sci Monit.

[18] Helthuis JHG, van Doormaal TPC, Hillen B, Bleys RLAW, Harteveld AA, Hendrikse J, van der Toorn A, Brozici M, Zwanenburg JJM, van der Zwan A (2019). Branching pattern of the cerebral arterial tree. Anat Rec.

[19] Huang X, Metaxas D, Chen T (2004) Metamorphs: deformable shape and texture models. In: Proceedings of the 2004 IEEE Computer Society Conference on Computer Vision and Pattern Recognition, 2004. CVPR 2004. IEEE, pp I-I

[20] Huttenlocher DP, Klanderman GA, Rucklidge WJ (1993). Comparing images using the Hausdorff distance. IEEE Trans Pattern Anal Mach Intell.

[21] Jin D, Saha PK (2013) A new fuzzy skeletonization algorithm and its applications to medical imaging. In: International Conference on Image Analysis and Processing. Springer, pp 662–671

[22] Johnson CO, Nguyen M, Roth GA, Nichols E, Alam T, Abate D, Abd-Allah F, Abdelalim A, Abraha HN, Abu-Rmeileh NM, Adebayo OM, Adeoye AM, Agarwal G, Agrawal S, Aichour AN, Aichour I, Aichour MTE, Alahdab F, Ali R, Alvis-Guzman N, Anber NH, Anjomshoa M, Arabloo J, Arauz A, Ärnlöv J, Arora A, Awasthi A, Banach M, Barboza MA, Barker-Collo SL, Bärnighausen TW, Basu S, Belachew AB, Belayneh YM, Bennett DA, Bensenor IM, Bhattacharyya K, Biadgo B, Bijani A, Bikbov B, Bin Sayeed MS, Butt ZA, Cahuana-Hurtado L, Carrero JJ, Carvalho F, Castañeda-Orjuela CA, Castro F, Catalá-López F, Chaiah Y, Chiang PP-C, Choi J-YJ, Christensen H, Chu D-T, Cortinovis M, Damasceno AAM, Dandona L, Dandona R, Daryani A, Davletov K, De Courten B, De La Cruz-Góngora V, Degefa MG, Dharmaratne SD, Diaz D, Dubey M, Duken EE, Edessa D, Endres M, Faraon EJA, Farzadfar F, Fernandes E, Fischer F, Flor LS, Ganji M, Gebre AK, Gebremichael TG, Geta B, Gezae KE, Gill PS, Gnedovskaya EV, Gómez-Dantés H, Goulart AC, Grosso G, Guo Y, Gupta R, Haj-Mirzaian A, Haj-Mirzaian A, Hamidi S, Hankey GJ, Hassen HY, Hay SI, Hegazy MI, Heidari B, Herial NA, Hosseini MA, Hostiuc S, Irvani SSN, Islam SMS, Jahanmehr N, Javanbakht M, Jha RP, Jonas JB, Jozwiak JJ, Jürisson M, Kahsay A, Kalani R, Kalkonde Y, Kamil TA, Kanchan T, Karch A, Karimi N, Karimi-Sari H, Kasaeian A, Kassa TD, Kazemeini H, Kefale AT, Khader YS, Khalil IA, Khan EA, Khang Y-H, Khubchandani J, Kim D, Kim YJ, Kisa A, Kivimäki M, Koyanagi A, Krishnamurthi RK, Kumar GA, Lafranconi A, Lewington S, Li S, Lo WD, Lopez AD, Lorkowski S, Lotufo PA, Mackay MT, Majdan M, Majdzadeh R, Majeed A, Malekzadeh R, Manafi N, Mansournia MA, Mehndiratta MM, Mehta V, Mengistu G, Meretoja A, Meretoja TJ, Miazgowski B, Miazgowski T, Miller TR, Mirrakhimov EM, Mohajer B, Mohammad Y, Mohammadoo-Khorasani M, Mohammed S, Mohebi F, Mokdad AH, Mokhayeri Y, Moradi G, Morawska L, Moreno Velásquez I, Mousavi SM, Muhammed OSS, Muruet W, Naderi M, Naghavi M, Naik G, Nascimento BR, Negoi RI, Nguyen CT, Nguyen LH, Nirayo YL, Norrving B, Noubiap JJ, Ofori-Asenso R, Ogbo FA, Olagunju AT, Olagunju TO, Owolabi MO, Pandian JD, Patel S, Perico N, Piradov MA, Polinder S, Postma MJ, Poustchi H, Prakash V, Qorbani M, Rafiei A, Rahim F, Rahimi K, Rahimi-Movaghar V, Rahman M, Rahman MA, Reis C, Remuzzi G, Renzaho AMN, Ricci S, Roberts NLS, Robinson SR, Roever L, Roshandel G, Sabbagh P, Safari H, Safari S, Safiri S, Sahebkar A, Salehi Zahabi S, Samy AM, Santalucia P, Santos IS, Santos JV, Santric Milicevic MM, Sartorius B, Sawant AR, Schutte AE, Sepanlou SG, Shafieesabet A, Shaikh MA, Shams-Beyranvand M, Sheikh A, Sheth KN, Shibuya K, Shigematsu M, Shin M-J, Shiue I, Siabani S, Sobaih BH, Sposato LA, Sutradhar I, Sylaja P, Szoeke CEI, Te Ao BJ, Temsah M-H, Temsah O, Thrift AG, Tonelli M, Topor-Madry R, Tran BX, Tran KB, Truelsen TC, Tsadik AG, Ullah I, Uthman OA, Vaduganathan M, Valdez PR, Vasankari TJ, Vasanthan R, Venketasubramanian N, Vosoughi K, Vu GT, Waheed Y, Weiderpass E, Weldegwergs KG, Westerman R, Wolfe CDA, Wondafrash DZ, Xu G, Yadollahpour A, Yamada T, Yatsuya H, Yimer EM, Yonemoto N, Yousefifard M, Yu C, Zaidi Z, Zamani M, Zarghi A, Zhang Y, Zodpey S, Feigin VL, Vos T, Murray CJL (2019). Global, regional, and national burden of stroke, 1990–2016: a systematic analysis for the global burden of disease study 2016. Lancet Neurol.

[23] Kozinska D, Tretiak J, Nissanov J, Ozturk C (1997). Multidimensional alignment using the Euclidean distance transform. Graph Models Image Process.

[24] Lesage D, Angelini ED, Bloch I, Funka-Lea G (2009). A review of 3D vessel lumen segmentation techniques: models, features and extraction schemes. Med Image Anal.

[25] Lugauer F, Zhang J, Zheng Y, Hornegger J, Kelm BM (2014) Improving accuracy in coronary lumen segmentation via explicit calcium exclusion, learning-based ray detection and surface optimization, Medical Imaging 2014: Image Processing. International Society for Optics and Photonics, p 90343U

[26] Masutani Y, Schiemann T, Höhne K-H (1998) Vascular shape segmentation and structure extraction using a shape-based region-growing model. In: International Conference on Medical Image Computing and Computer-Assisted Intervention. Springer, pp 1242–1249

[27] McCormick MM, Liu X, Ibanez L, Jomier J, Marion C (2014). ITK: enabling reproducible research and open science. Front Neuroinform.

[28] Morillon B, Lehongre K, Frackowiak RS, Ducorps A, Kleinschmidt A, Poeppel D, Giraud A-L (2010). Neurophysiological origin of human brain asymmetry for speech and language. Proc Natl Acad Sci.

[29] Nealen A, Igarashi T, Sorkine O, Alexa M (2006) Laplacian mesh optimization. In: Proceedings of the 4th international conference on computer graphics and interactive techniques in Australasia and Southeast Asia, pp 381–389

[30] Németh G, Kardos P, Palágyi K (2011). Thinning combined with iteration-by-iteration smoothing for 3D binary images. Graph Models.

[31] Nowinski WL, Volkau I, Marchenko Y, Thirunavuukarasuu A, Ng TT, Runge VM (2009). A 3D model of human cerebrovasculature derived from 3T magnetic resonance angiography. Neuroinformatics.

[32] Pawlak MA, Krejza J, Rudzinski W, Kwiatkowski JL, Ichord R, Jawad AF, Tomaszewski M, Melhem ER (2009). Sickle cell disease: ratio of blood flow velocity of intracranial to extracranial cerebral arteries—initial experience. Radiology.

[33] Piccinelli M, Veneziani A, Steinman DA, Remuzzi A, Antiga L (2009). A framework for geometric analysis of vascular structures: application to cerebral aneurysms. IEEE Trans Med Imaging.

[34] Pijewska E, Sylwestrzak M, Gorczynska I, Tamborski S, Pawlak MA, Szkulmowski M (2020). Blood flow rate estimation in optic disc capillaries and vessels using Doppler optical coherence tomography with 3D fast phase unwrapping. Biomed Opt Express.

[35] Robben D, Turetken E, Sunaert S, Thijs V, Wilms G, Fua P, Maes F, Suetens P (2014) Simultaneous segmentation and anatomical labeling of the cerebral vasculature. Medical image computing and computer-assisted intervention : MICCAI ... International Conference on Medical Image Computing and Computer-Assisted Intervention 17:307–314 10.1007/978-3-319-10404-1_39 25333132

[36] Schroeder W, Martin K, Lorensen B (2006) The Visualization Toolkit, 4th edKitware. New York

[37] Shahzad R, Kirişli H, Metz C, Tang H, Schaap M, van Vliet L, Niessen W, van Walsum T (2013). Automatic segmentation, detection and quantification of coronary artery stenoses on CTA. Int J Cardiovasc Imaging.

[38] Stamatelos SK, Bhargava A, Kim E, Popel AS, Pathak AP (2019). Tumor ensemble-based modeling and visualization of emergent angiogenic heterogeneity in breast cancer. Sci Rep.

[39] Turetken E, Benmansour F, Andres B, Pfister H, Fua P (2013) Reconstructing loopy curvilinear structures using integer programming. In: Proceedings of the IEEE Conference on Computer Vision and Pattern Recognition, pp 1822–1829

[40] Verscheure L, Peyrodie L, Makni N, Betrouni N, Maouche S, Vermandel M (2010) Dijkstra’s algorithm applied to 3D skeletonization of the brain vascular tree: Evaluation and application to symbolic, 2010 Annual International Conference of the IEEE Engineering in Medicine and Biology. IEEE, pp 3081–3084 10.1109/IEMBS.2010.5626112 21095739

[41] Vingerhoets G, Stroobant N (1999). Lateralization of cerebral blood flow velocity changes during cognitive tasks: a simultaneous bilateral transcranial Doppler study. Stroke.

[42] Volkau I, Ng TT, Marchenko Y, Nowinski WL (2008). On geometric modeling of the human intracranial venous system. IEEE Trans Med Imaging.

[43] Wang C, Moreno R, Smedby Ö (2012) Vessel segmentation using implicit model-guided level sets, MICCAI Workshop” 3D Cardiovascular Imaging: a MICCAI segmentation Challenge”, Nice France, 1st of October 2012

[44] Wink O, Niessen WJ, Viergever MA (2000). Fast delineation and visualization of vessels in 3-D angiographic images. IEEE Trans Med Imaging.

[45] Yim PJ, Cebral JJ, Mullick R, Marcos HB, Choyke PL (2001). Vessel surface reconstruction with a tubular deformable model. IEEE Trans Med Imaging.

[46] Zhou H, Min JK, Xiong G (2016) Implicit tubular surface generation guided by centerline. arXiv preprint arXiv:1606.03014

