# Correction to: Customizable tubular model for n‑furcating blood vessels and its application to 3D reconstruction of the cerebrovascular system

**DOI:** 10.1007/s11517-023-02796-0

**Published:** 2023-02-27

**Authors:** Michal Chlebiej, Anna Zurada, Jerzy Gielecki, Mikolaj A. Pawlak, Maciej Szkulmowski

**Affiliations:** 1 Faculty of Mathematics and Computer Science, Nicolaus Copernicus University in Toruń, Chopina 12/18, 87‑100, Torun, Poland; 2 Department of Radiology, Collegium Medicum, School of Medicine, University of Warmia and Mazury, Olsztyn, Poland; 3 Department of Anatomy, Collegium Medicum, University of Warmia and Mazury, Olsztyn, Poland; 4 Department of Neurology and Cerebrovascular Disorders, Poznan University of Medical Sciences, Fredry 10, 61‑701, Poznan, Poland; 5 Department of Clinical Genetics, Erasmus MC, PO Box 2040, 3000 CA Rotterdam, The Netherlands; 6 Faculty of Physics, Astronomy and Informatics, Nicolaus Copernicus University in Toruń, Grudziadzka 5, 87‑100, Torun, Poland

**Correction to: Medical & Biological Engineering & Computing**

**https://doi.org/10.1007/s11517-022-02735-5**

The original article contained a mistake.

Affiliation of Maciej Szkulmowski is incorrect. He is only affiliated with affiliation 6. The affiliation address of professor Jerzy Gielecki has also been corrected (after correction: Department of Anatomy, Collegium Medicum, University of Warmia and Mazury, Olsztyn, Poland) .  In the final paper, all affiliations have been renumbered in ascending order.

The original article has been corrected.

